# Targeting cholesterol impairs cell invasion of all breast cancer types

**DOI:** 10.1186/s12935-023-03206-z

**Published:** 2024-01-10

**Authors:** Mauriane Maja, Marie Verfaillie, Patrick Van Der Smissen, Patrick Henriet, Christophe E. Pierreux, Nor Eddine Sounni, Donatienne Tyteca

**Affiliations:** 1 CELL Unit and PICT Imaging Platform, de Duve Institute, UCLouvain, 1200 Brussels, Belgium; 2 Laboratory of Tumor and Development Biology, GIGA-Cancer, University of Liège, 4000 Liège, Belgium

**Keywords:** Cholesterol submicrometric domains, Breast cancer cell lines, Matrigel invasion, 3D spheroid growth, 3D spheroid invasion, Lipid droplets, Cortactin, MMP inhibition

## Abstract

**Background:**

Breast cancer clinical outcome relies on its intrinsic molecular subtype and mortality is almost exclusively due to metastasis, whose mechanism remains unclear. We recently revealed the specific contribution of plasma membrane cholesterol to the invasion of malignant MCF10CAIa but not premalignant MCF10AT and normal MCF10A cell lines in 2D, through invadopodia formation and extracellular matrix (ECM) degradation. In the present study, we address the impact of breast cancer subtypes, mutations and aggressiveness on cholesterol implication in breast cancer cell invasion and 3D spheroid invasion and growth.

**Methods:**

We used nine breast cancer cell lines grouped in four subtypes matching breast tumor classification. Four of these cell lines were also used to generate 3D spheroids. These cell lines were compared for cell invasion in 2D and 3D, spheroid growth in 3D, gelatin degradation, cortactin expression, activation and subcellular distribution as well as cell surface cholesterol distribution and lipid droplets. The effect of plasma membrane cholesterol depletion on all these parameters was determined in parallel and systematically compared with the impact of global matrix metalloproteinase (MMP) inhibition.

**Results:**

The six invasive cell lines in 2D were sensitive to partial cholesterol depletion, independently of their subtype, aggressiveness or mutation. Nevertheless, the effect was stronger in the three cell lines able to degrade gelatin. 3D spheroid invasion was also reduced after cholesterol depletion in all breast cancer subtypes tested. Notably, targeting cholesterol was more powerful than MMP inhibition in reducing invasion in both 2D and 3D culture models. Moreover, cholesterol depletion in the six invasive cell lines impaired cortactin distribution in the perinuclear region where invadopodia localized. Breast cancer cell line aggressiveness relied on cholesterol-enriched domains at the ECM-free side and intracellular lipid droplets. Furthermore, the three gelatin-degrading cell lines were characterized by increased cholesterol-enriched submicrometric domains at their ECM-contact side.

**Conclusion:**

Together, our data suggest cell surface cholesterol combined with lipid droplet labeling as a breast cancer cell aggressiveness marker. They also open the way to test other cholesterol-targeting drugs in more complex models to further evaluate whether cholesterol could represent a strategy in breast cancer therapy.

**Supplementary Information:**

The online version containssupplementary material available at 10.1186/s12935-023-03206-z.

## Introduction

Breast cancer is the most diagnosed cancer worldwide accounting for 11.7% of the total number of new cases in 2020 and the leading cause of cancer death in women [1]. The clinical outcome of breast cancer relies on its intrinsic molecular subtype. In fact, this heterogeneous neoplasm has been classified into 5 subtypes based on gene expression profile: (1) luminal A-like, (2) luminal B-like HER2−, (3) luminal B-like HER2+, (4) HER2-enriched, and (5) basal-like triple-negative (TN); all of these have major differences in incidence, biomarkers, treatment strategies, aggressiveness and prognosis [2]. In general, TN breast cancer (TNBC) is associated with a worse prognosis than other subtypes due to earlier recurrences and metastases [3]. Currently, breast cancer mortality is almost exclusively due to metastasis, although it remains unclear how tumor cell features lead to metastasis [4].

Metastasis is a multistep process by which cancer cells leave the primary tumor, circulate in the bloodstream, and reach a distant site to develop a secondary tumor. Invasion of adjacent tissue, a key feature of the metastatic cascade, is governed by cancer cell motility and plasticity. The cytoskeleton has been shown to contribute to cancer cell deformability and mechanical response during invasion [5, 6]. Recently, cholesterol (chol) has been the center of many investigations for its implication in tumor cell survival, proliferation, migration and invasion [7]. Chol content is often described as altered in cancer cells due to a reprogrammed metabolism [8]. In breast cancer for instance, high expression of chol biosynthesis-related genes correlates with aggressive subtypes and can be inhibited to reduce breast cancer cell invasion in vitro [9–11].

In support of the deregulation of the plasma membrane (PM) chol in breast cancer metastasis, we previously showed that the malignant mammary cell line MCF10CAIa exhibits a higher surface chol content together with a stiffening of the PM, compared to isogenic and non-tumorigenic MCF10A cells [12]. This PM chol is distributed in submicrometric domains, which are specifically increased in abundance in the malignant MCF10CAIa cells and contribute to cell invasion through invadopodia formation and ECM degradation [13]. Accordingly, other studies have shown that lipid rafts and caveolae are required for the biogenesis and proteolytic activity of invadopodia in the TNBC cell line MDA-MB-231 cultured in 2D [14, 15]. Invadopodia are F-actin-enriched membrane protrusions regulated by cortactin and specialized in extracellular matrix (ECM) degradation by matrix metalloproteinases (MMP), some of which are known to be upregulated in breast cancer [16, 17]. Accordingly, PM chol depletion reduces TNBC cell line migration [18, 19]. Conversely, several studies reported that lower PM chol levels promote breast cancer cell migration by increasing membrane fluidity and deformability [20, 21]. Thus, further investigations on the role of chol in 3D models and in different breast cancer subtypes with different mutations are required before paving the way to use chol as a therapeutic target.

Therefore, we here address the impact of breast cancer subtypes, mutations and aggressiveness on PM chol implication in breast cancer cell invasion. Nine breast cancer cell lines grouped in four subtypes matching breast tumor classification were cultured in 2D. Four of these cell lines were also used to generate 3D spheroids. These cell lines were compared for cell invasion in 2D and 3D, spheroid growth in 3D, gelatin degradation, cortactin expression, activation and subcellular distribution as well as cell surface chol distribution and lipid droplets. We also evaluated the effect of chol-targeting molecules on the above parameters using methyl-β-cyclodextrin (mβCD). This choice was based on our previous observation that the mammary malignant cell line MCF10CAIa exhibits a higher abundance of surface chol-enriched domains than the premalignant MCF10AT and normal MCF10A cell lines and that chol depletion by mβCD specifically reduces chol-enriched domains and invasion of the malignant cell line. In parallel, we explored whether the effect of chol depletion could be more powerful than broad-spectrum MMP inhibitors, revealed in the past as promising in vitro but that failed in clinical trials mainly due to lack of inhibitor specificity [22, 23].

We revealed for the first time the contribution and possibility to target PM chol to reduce breast cancer invasion whatever the subtype or aggressiveness while MMP inhibition was less powerful. We also demonstrated the superiority of 3D spheroids over 2D culture for cell invasive capacity and impact of chol depletion but not of MMP inhibition, also independently of breast cancer cell subtype, aggressiveness and invasion mechanism. We also showed that chol depletion affected cortactin distribution in the invasion area at the benefit of the cell edges in all invasive cell lines.

## Materials and methods

### 2D Cell culture and chemical treatments

All cell lines were grown in medium composed of Dulbecco's Modified Eagle Medium (DMEM) supplemented with 10% Fetal Bovine Serum (FBS), penicillin (100 U/mL) and streptomycin (100 µg/mL) (i.e. complete medium) in a humidified atmosphere with 5% CO_2_ at 37 °C. To partially deplete chol, cells were pre-incubated in a serum-free medium containing 2 mM methyl-β-cyclodextrin (mβCD; Sigma-Aldrich #C4555) for 2 h at 37 °C (except otherwise stated). Following chol depletion, cells were either directly collected for western blotting, tested for surface chol and lipid droplets, or incubated in serum-containing medium during 12 h for invasion assay, 6–12 h for gelatin degradation assay or 4 h for cell immunofluorescence, as explained below. To inhibit MMP activity, cells were maintained during the whole experiment in a serum-free medium containing 10 µM GM6001 MMP inhibitor (Enzo Life Sciences #BML-EI300-0001) at 37 °C.

### 2D transwell invasion assay and quantification

Transwell invasion assay was performed as previously described [13]. Briefly, 3.5 × 10^4^ cells were seeded on top of a 2 mg/mL ECM gel (Sigma-Aldrich #E6909) coated insert (8 µm pore size, Greiner #665638), and were either serum-starved combined or not with mβCD for 2 h and then allowed to invade for 12 h or serum-starved for 2 h and then treated with GM6001 and allowed to invade during 12 h. The upper chamber was filled with serum-free medium whereas the bottom chamber was filled with serum-free medium or 10% serum-containing medium to assess spontaneous and total invasion, respectively. Non-invading cells were removed from the upper chamber with cotton swab and images of invading cells were captured with a wide-field fluorescence microscope Observer Z1 (20× objective). Images were analyzed with ZEN 2.6 software (Blue edition Zeiss). Oriented invasion index was calculated by subtracting the number of spontaneously invading cells (serum-free medium in the bottom chamber) from the total number of invading cells (determined in the presence of serum in the bottom chamber).

### Gelatin degradation assay and quantification

Gelatin degradation assay was performed as previously described [13]. In brief, cells in suspension were added to wells containing equilibrated Oregon Green™ 488 conjugate (ThermoFisher Scientific #G13186) gelatin-coated coverslips, either serum-starved combined or not with mβCD for 2 h and then incubated in serum-containing medium for 6–12 h  or serum-starved for 2 h and then incubated in GM6001-supplemented medium for 6–12 h. Cells were fixed in 4% paraformaldehyde for 30 min, permeabilized with 0.1% Triton X-100 in PBS for 15 min, blocked with 5% Normal Goat Serum in PBS for 1 h, all steps at room temperature, immunolabeled with anti-Cortactin (Cell Signaling Technology #3503, 1:200) overnight at 4 °C, and stained with fluorescent secondary antibodies (ThermoFisher Scientific, 1:1000), Alexa Fluor™ 647 Phalloidin (ThermoFisher Scientific #A22287, 1:200) and Hoechst 33258 (Abcam #Ab228550, 1:1000) for 1 h at room temperature in the dark. Coverslips were then mounted with Dako and examined with a Zeiss Cell Observer Spinning Disk (COSD) confocal microscope using a plan-Apochromat 63 × NA 1.4 water immersion objective and the same settings for illumination. Quantification of total gelatin degradation area per total cell area was done using ImageJ/Fiji [13].

### Cell immunofluorescence

Cells were seeded on fibronectin-precoated (Gibco #33010018) coverslips, serum-starved combined or not with mβCD for 2 h, then incubated in serum-containing medium for 4 h to allow invadopodia formation or serum-starved for 2 h and then incubated in GM6001-supplemented medium for 4 h. Cells were immunolabeled as described above with anti-Cortactin, anti-GM130 (BD Biosciences #610823, 1/100), anti-Paxillin (Millipore #05-417, 1:200), anti-Tubulin (Sigma-Aldrich #T6199, 1:100) and/or stained for F-actin and nuclei with Alexa Fluor™ 647 Phalloidin and Hoechst 33258. All coverslips were examined with a COSD confocal microscope using a plan-Apochromat 100 × NA 1.4 oil immersion objective.

### Western blotting and quantification

Cells were starved or treated with mβCD for 2 h or with GM6001 for 6 h, washed in PBS, lysed in cold RIPA lysis buffer (150 mM NaCl, 0.5% sodium deoxycholate, 50 mM Tris, 0.1% SDS, 1% Triton X-100, 1 mM PMSF, cOmplete™ Protease Inhibitor Cocktail) for 25 min, then sonicated for 2 × 15 s and centrifuged 5 min at 10.500 rpm at 4 °C. Protein concentration was measured by the bicinchoninic acid (BCA) method. Cell lysates were diluted in loading buffer (0.25 M Tris–HCl, pH 6.8, 10% SDS, 20% glycerol, 0.005% bromophenol blue) containing 5 mM Dithiothreitol (DTT) and boiled for 5 min. Equal protein amounts were separated by SDS-PAGE (Running Buffer: 0.025 M Tris, 0.192 M glycine, 0.1% SDS) on 8 or 10% homemade polyacrylamide gels and transferred onto PVDF membrane. Membranes were blocked for 2 h in Tris Buffered Saline Tween (TBST)-5% milk and then incubated overnight at 4 °C with anti-Cortactin (1:1000), anti-phospho-Cortactin (Cell Signaling Technology #4569, 1:1000), anti-Estrogen Receptor α (Santa Cruz #sc-8002, 1:200), anti-Progesterone Receptor (Santa Cruz #sc-166169, 1:200), anti-HER2/ErbB2 (Cell Signaling Technology #2165, 1:1000), anti-E-cadherin (BD Biosciences #610181, 1:2500), anti-Akt (Cell Signaling Technology #9272, 1:1000), anti-phospho-Akt (Cell Signaling Technology #4060, 1:2000) or anti-GAPDH (Millipore #ABS16, 1:1000) primary antibodies, washed and then incubated for 1 h at room temperature with horseradish peroxidase-conjugated secondary antibodies, all diluted in TBST-5% milk. Immunoreactive bands were revealed using Super Signal Chemiluminescent Substrate (ThermoFisher Scientific) and images were acquired using Fusion Solo S (Vilber Lourmat, Collegien, France). Quantification of band intensity was performed using ImageJ/Fiji and normalized by GAPDH band intensity.

### Live cell imaging of cholesterol and lipid droplets and quantification

Cells were seeded on fibronectin-precoated coverslips, starved or treated with mßCD for 2 h or with GM6001 for 4 h, washed 2 times with DMEM at room temperature and then labeled for surface chol or lipid droplets. For surface chol labeling, cells were incubated with 1 µM mCherry-Theta toxin fragment in fatty acid-free Bovine Serum Albumin (1 mg/mL; Sigma-Aldrich #A8806) for 20 min at 4 °C. Expression, purification, biochemical characterization and storage of the probe were performed as described in [24]. For lipid droplet labeling, cells were incubated with 1.8 µM BODIPY^493/503^ (Thermo Fisher Scientific #D3922) in fatty acid-free Bovine Serum Albumin (1 mg/mL) for 20 min at 37 °C. Labeled cells were then washed 2 times with DMEM at room temperature. The coverslips were then transferred in medium-filled LabTek chambers and observed with the COSD confocal microscope using a plan-Apochromat 63 × NA 1.4 water immersion objective. X–Y confocal images and/or z-stacks were acquired with the same laser settings and exposure time. Chol distribution at the ECM-free cell side was quantified on z-stacks using ImageJ/Fiji as previously [13]. Data are presented as ECM-free side Theta fluorescence intensity. Chol distribution at the ECM-contact side was estimated through a score: 0, absence of chol; 0.5, low chol; 1, chol present but lower than at the ECM-free side; 2, high chol comparable to chol content at the ECM-free side. Quantification of the total cell and specific lipid droplet fluorescence signals was done using ZEN 3.3 software (Zeiss) by a homemade macro. Background was estimated in regions where no signal was expected, especially in the nucleus. All experimental conditions were compared to ensure that no signal saturation was present in the specific labeled structures. To exclude background, a window of segmentation was chosen for whole cells between 60 and 4095 and for lipid droplets between 300 and 4095. Data are presented as total BODIPY^493/503^ fluorescence area or lipid droplet area occupancy.

### 3D tumor spheroid formation and growth assay and quantification

Cells were suspended in complete medium to a final concentration of 5–10 × 10^3^ cells/mL and 200 µL of this suspension was seeded in 96-well ultra-low attachment plates (Corning #7007) and centrifuged at 250 rpm for 5 min. After 2–3 days of growth at 37 °C, 100 µL of medium was removed and replaced by 100 µL of mix at 2 × final assay concentration containing complete medium supplemented or not with 2.5 mM mβCD or 60 µM GM6001 (unless otherwise stated, final assay concentration of 1.25 mM mβCD or 30 µM GM6001) and the plates were placed at 37 °C. Spheroid growth was then monitored and imaged every 24 h for 7 days with the wide-field fluorescence microscope Observer Z1 (10× objective). Quantification of spheroid area growth was performed with Arivis4D software (Zeiss) by a standard delivered pipeline in the software: “Detect Big Structures Auto”. Spheroid segmentation was best visualized by the Otsu Threshold method (standard in the software). The selected area for spheroids was comprised between 15.000 and 800.000 µm^2^. Diameter and projected area were quantified and data are presented as percent increase of day 0 spheroid area.

### 3D tumor spheroid invasion assay and quantification

Spheroids were formed as described above with 1 × 10^3^ cells per well in complete medium. After 3 days of growth, 100 µL of medium was removed and replaced by 100 µL of mix at 2 × final assay concentration containing 4 mg/mL Matrigel (Corning #356234) supplemented or not with 2.5 mM mβCD or 60 µM GM6001 (unless otherwise stated, final assay concentration of 2 mg/mL Matrigel, 1.25 mM mβCD or 30 µM GM6001) and allowed to polymerize for 1 h at 37 °C. Then, 50 µL of complete medium containing 1.25 mM mβCD or 30 µM GM6001 at 1 × final assay concentration was added per well and the plates were placed at 37 °C. Spheroid invasion was monitored and imaged every 24 h for 7 days with the wide-field fluorescence microscope Observer Z1 (10 × objective). Three types of quantification were made. First, spheroid area after invasion was determined using Arivis4D software as described above for spheroids without protrusions, or ImageJ/Fiji for protrusions-forming spheroids. For the latter, images were converted to 8-bit grayscale, manual threshold was applied to segment spheroids, images were converted to mask, holes were filled, and then total spheroid area was measured. Second, spheroid 3D invasion potential was calculated by subtracting the spheroid area without Matrigel from the area with Matrigel. Data are presented as percent increase of day 0 or untreated spheroid area. Third, mean length of the 5 longest protrusions from the outer rim of the dense spheroid core was measured using ImageJ/Fiji.

### Data presentation and statistical analyses

Each cell line was associated with a color code (Additional file 1: Table S1). Graphs and statistical analyses were performed with GraphPad Prism Software Version 8.0.2. For invasion assay, gelatin degradation assay, western blotting and PM chol, data are expressed as means of n independent experiments ± SD. For lipid droplets abundance, data are expressed as means of n images from 1 experiment ± SD. For spheroid growth and invasion assays, data are expressed as means of n spheroids from ≥ 2 independent experiments ± SD. For all experiments, statistical tests were performed only when n ≥ 3. To compare the cell lines, parametrical ordinary one-way ANOVA followed by suitable post-hoc comparison test was done. To compare the effect of one treatment with a hypothetical mean of 100% representing the control, parametrical one sample t test or non-parametrical Wilcoxon signed-rank test were performed. ns not significant, ^*^p < 0.05, ^**^p < 0.01, ^***^p < 0.001, ^****^p < 0.0001.

## Results

### HER2-enriched and basal-like triple-negative cell lines are able of invasion in 2D and differ by their aggressiveness score

The 9 breast cancer cell lines used in this study are representative of 4 subtypes determined based on their tumor origin, hormone receptor status (expression profile of estrogen receptor, progesterone receptor and HER2) and mutations: (1) luminal A-like (T-47D and ZR-75-1), (2) luminal B-like HER2+ (SK-BR-3), (3) HER2-enriched (HCC1569 and HCC1954) and (4) basal-like TN (MDA-MB-468, Hs-578T, BT-549 and MDA-MB-231) (Additional file 1: Table S1). These cell lines effectively expressed the above molecular makers (Additional file 1: Fig. S1A–C) and differed by their aggressiveness score determined based on: (1) cell morphology (Additional file 1: Fig. S2), (2) epithelial-mesenchymal transition (E-cadherin expression; Additional file 1: Fig. S1D), (3) proliferation rate (estimated from cell culture), (4) survival (Akt phosphorylation; Additional file 1: Fig. S1E, F), and (5) invasive capacity (oriented, total and spontaneous invasion using Matrigel-coated Transwell; Additional file 1: Fig. S3A–C). Cell line comparison for the latter parameter indicated that the 3 luminal cell lines were not capable of invasion, HCC1569 were barely invasive and HCC1954, MDA-MB-468 and MDA-MB-231 were highly invasive but twice less than Hs-578T and BT-549 (Additional file 1: Fig. S3A–C). Altogether, low aggressiveness scores were attributed to T-47D, ZR-75-1 and HCC1569, intermediate scores to SK-BR-3, HCC1954 and MDA-MB-468, and high scores to basal-like TN Hs-578T, BT-549 and MDA-MB-231, with Hs-578T and BT-549 being the most aggressive cell lines in this study (Additional file 1: Table S2).

### Cholesterol depletion reduces oriented invasion of the 6 invasive cell lines but to a differential extent

**Fig. 1:**
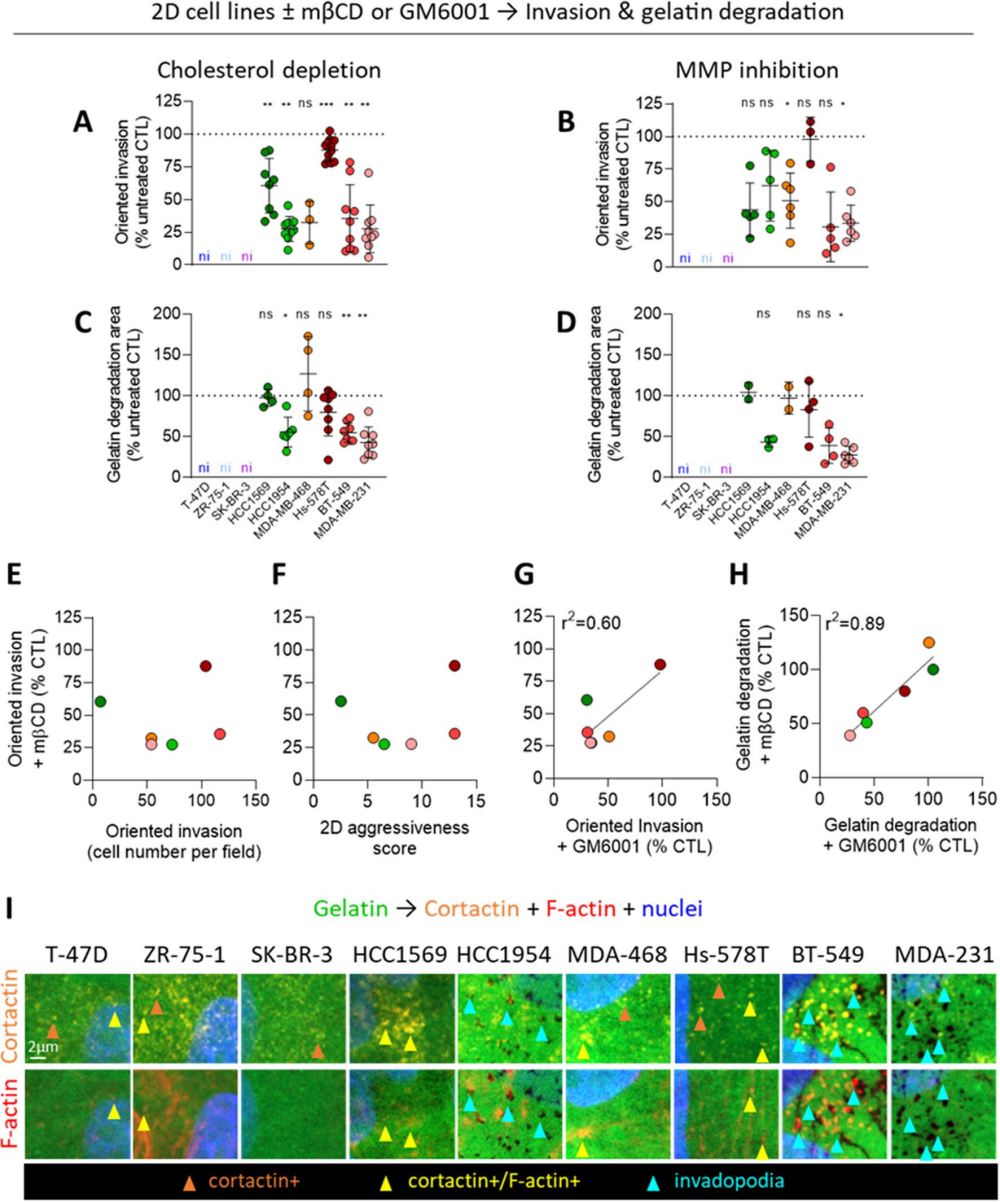
Cholesterol depletion decreases invasion of the 6 invasive cell lines in 2D. Nine cell lines were either serum-starved combined or not with 2 mM mβCD (chol depletion) for 2 h (**A, C**) or treated with 10 µM GM6001 (MMP inhibition) during the whole experiment (**B, D**) and then tested for oriented invasion (**A**, **B**) and gelatin degradation (**C, D, I**). **A**, **B** Quantification of oriented cell invasion in Matrigel-coated Transwell toward 10% serum for 12 h upon mβCD or GM6001 (n = 3–12 Transwell from 2–5 independent experiments and n = 3–6 Transwell from 1–3 independent experiments, respectively). The oriented invasion index was calculated by subtracting the number of spontaneously invading cells toward 0% serum. ni, non-invasive. For determination of invasion capacity, see Fig. S3A. **C**, **D** Quantification of gelatin degradation potential of cell lines serum-starved combined or not with mβCD for 2 h (**C**) then stimulated for 6–12 h with serum-containing medium or serum-starved for 2 h and then incubated in GM6001-supplemented medium for 6–12 h (**D**; 188–669 cells from n = 22–78 images from 2–5 independent experiments and 284–699 cells from n = 12–62 images from 1–4 independent experiments, respectively). Wilcoxon signed-rank test (**A**–**D**). **E**–**H** Relations between the oriented invasion of 6 cell lines treated with mβCD and number of invading cells (**E**), 2D aggressiveness score (**F**) or oriented invasion upon GM6001 (**G**), or between gelatin degradation potential upon both drugs (**H**). **I** Representative confocal images of gelatin degradation areas (black areas) of the 9 cell lines (immuno) labeled with anti-Cortactin, Phalloidin (F-actin) and Hoechst (nuclei). Orange, yellow and blue arrowheads, cortactin+ structures, cortactin/F-actin+ structures and cortactin/F-actin+ structures associated with degradation areas, respectively (n = 1–5). For enlarged views of the 3 degrading cell lines, please refer to Additional file 1: Fig. S5B

To evaluate the potential implication of chol in the invasion of the 6 most invasive cell lines (i.e. the HER2-enriched and basal-like TN ones), chol was partially depleted by a 2 h incubation with 2 mM mβCD, as previously [13]. The spontaneous invasion in Transwell without serum in the bottom chamber was poorly affected by this treatment, except in HCC1569 and BT-549 for which a ~ 35% reduction was observed (Additional file 1: Fig. S3E). In contrast, total and oriented invasion indexes towards serum in the bottom chamber were decreased in all the cell lines although to different extent, the Hs-578T showing the weakest reduction (Fig. 1A, Additional file 1: Fig. S3D). This weaker decrease could partially result from a limited chol depletion as 4 mM mβCD decreased invasion to a higher extent (Additional file 1: Fig. S4A). Nevertheless, the decrease remained weaker than in other cell lines and a longer incubation time did not reach a stronger effect. We hypothesized that Hs-578T used other invasion mechanisms than the other cell lines. Accordingly, Hs-578T invasion was not affected by global inhibition of MMP activity with GM6001, in contrast to the other cell lines for which a similar effect of chol depletion and MMP inhibition was obtained and reflected by the positive correlation between oriented invasion extents upon mβCD and GM6001 (Fig. 1B, G). Moreover, Hs-578T, but also HCC1569 and MDA-MB-468, were not able to degrade Oregon Green gelatin and were sensitive neither to mβCD nor to GM6001 (Fig. 1C, D, Additional file 1: Fig. S5A). In contrast, HCC1954, BT-549 and MDA-MB-231 were capable of gelatin degradation, basal-like TN BT-549 cells exhibiting the highest degradation potential, and were sensitive to both chol depletion and MMP inhibition (Fig. 1C, D, Additional file 1: Fig. S5).

Altogether, these data showed that chol depletion reduced (i) spontaneous invasion of 2 cell lines while MMP inhibition did not (Additional file 1: Fig. S3E, G); (ii) oriented invasion of the 6 invasive cell lines whatever their invasion capacity or aggressiveness but to a lower extent in Hs-578T and more efficiently than MMP inhibition (Fig. 1A, B, E, F); and (iii) gelatin degradation of the 3 cell lines able to do it, like MMP inhibition (Fig. 1C, D). Importantly, while gelatin degradation potentials correlated very well upon either treatment, the correlation was weaker for cell invasion (Fig. 1G, H), which could suggest different invasion mechanisms.

### Cell lines differ by cortactin distribution but not activation

**Fig. 2:**
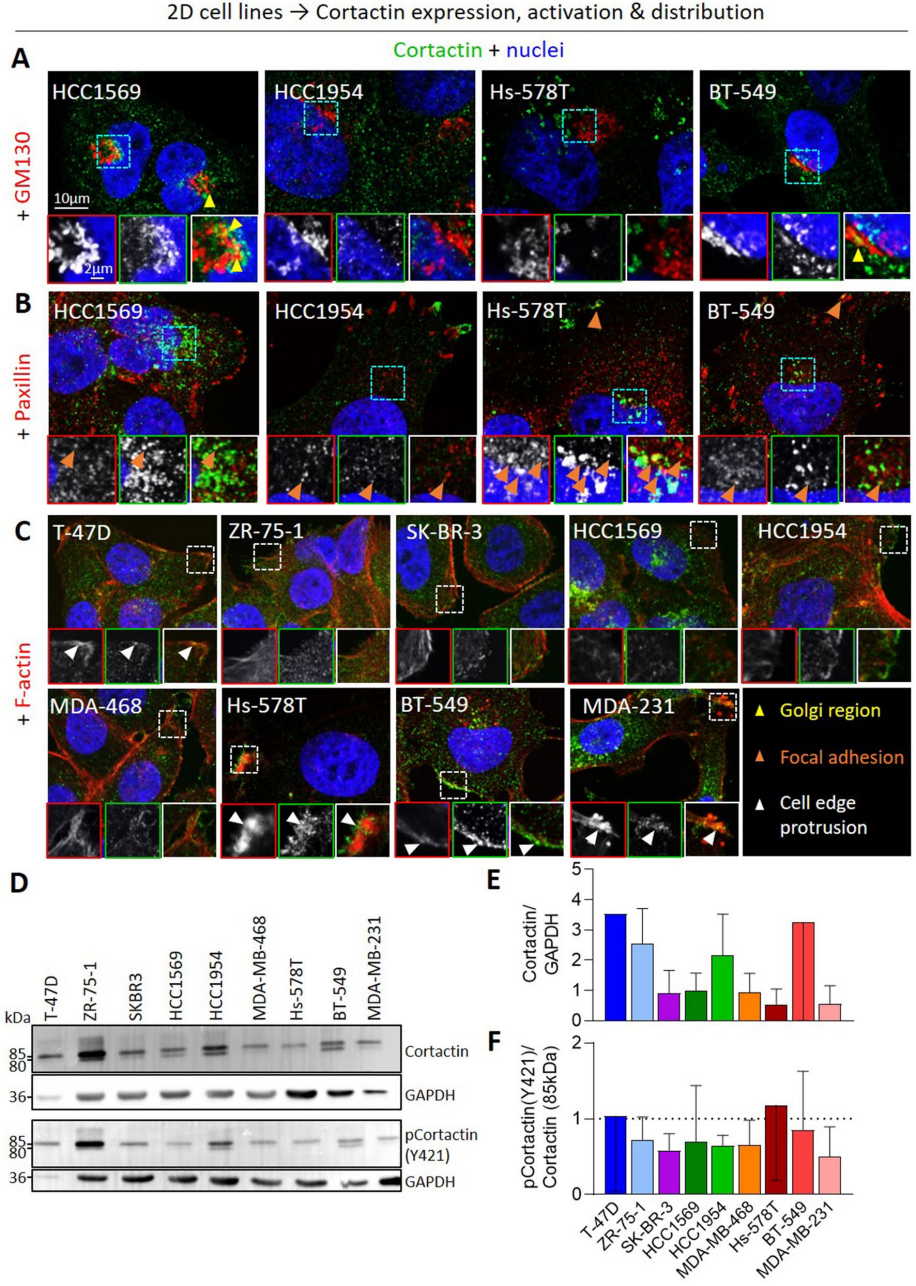
The 9 cell lines differ by cortactin subcellular localization but not by its phosphorylation extent. Cell lines were tested for cortactin distribution by confocal microscopy (**A**–**C**) and expression and activation by Western blots (**D**–**F**). **A**–**C** Confocal images of cell lines plated on fibronectin-coated coverslips, serum-starved for 2 h, then stimulated 4 h with serum-containing medium and (immuno)labeled with anti-Cortactin and anti-GM130 (Golgi; **A**), anti-Paxillin (focal adhesions; **B**) or Phalloidin (F-actin; **C**) and Hoechst (nuclei). Blue and white insets, perinuclear and edge regions respectively. Yellow, orange and white arrowheads, cortactin distribution in Golgi region, focal adhesions and cell edge protrusions, respectively (n = 1–3). **D** Western blots of cortactin and phosphorylated cortactin (pCortactin Y421) with both isomers at 80 kDa and 85 kDa in the 9 cell lines. GAPDH was used as loading control. **E**, **F** Quantification of total cortactin (80 + 85 kDa forms) protein abundance (**E**) and phosphorylation level of the 85 kDa form (ratio of pCortactinY421/cortactin; **F**) in the 9 cell lines (n = 2–6)

To address this possibility, cells on fluorescent gelatin were labeled for cortactin and F-actin to reveal invadopodia [13]. As expected, the three mβCD- and GM6001-sensitive and gelatin-degrading cell lines (HCC1954, BT-549 and MDA-MB-231) showed a co-enrichment of cortactin and F-actin coinciding with, or near, gelatin degradation areas (Fig. 1I black holes), indicating the presence of invadopodia (Fig. 1I blue arrowheads, Additional file 1: Fig. S5B for enlarged views). In the other 6 cell lines, cortactin was also localized in the perinuclear region but either not associated with gelatin degradation (yellow arrowheads) or even not colocalized with F-actin (orange arrowheads). Double labeling with GM130 revealed that the cortactin-positive structures were in very close proximity to the Golgi apparatus in HCC1569 and BT-549 (Fig. 2A yellow arrowheads), the two cell lines showing a reduced spontaneous invasion upon chol depletion. In contrast, the cortactin-positive structures observed in HCC1954 and Hs-578T did not colocalize with GM130 but partially with focal adhesions labeled for Paxillin (Fig. 2A, B orange arrowheads). Cortactin was also found at the cell edges particularly in T-47D and the 3 most aggressive TN cell lines, as revealed by co-labeling for F-actin with phalloidin (Fig. 2C white arrowheads). Altogether, these data indicated that cortactin was associated with invadopodia, Golgi and/or focal adhesions in the perinuclear region depending on the cell line but also with cell edge protrusions, especially in the 3 most aggressive basal-like TN cell lines.

To then evaluate whether this differential distribution could be associated with differential cortactin expression and/or activation, we performed western blotting against total and phosphorylated cortactin on tyrosine residue 421, a pro-migratory event associated with cancer and poor prognosis [25]. Two bands at 80 and 85 kDa were observed (Fig. 2D), in agreement with [26]. These bands could correspond to two cortactin conformational isomers, the “closed” 80 kDa state which prevents phosphorylation and the “open” 85 kDa state prone to phosphorylation. Quantification indicated that cortactin was expressed (Fig. 2D, E) and displayed a similar phosphorylation status at Y421 in all cell lines (Fig. 2D, F).

### Cholesterol depletion partially delocalizes cortactin from perinuclear region to cell edges and induces the formation of small non-polarized protrusions in the 3 gelatin-degrading cell lines

**Fig. 3:**
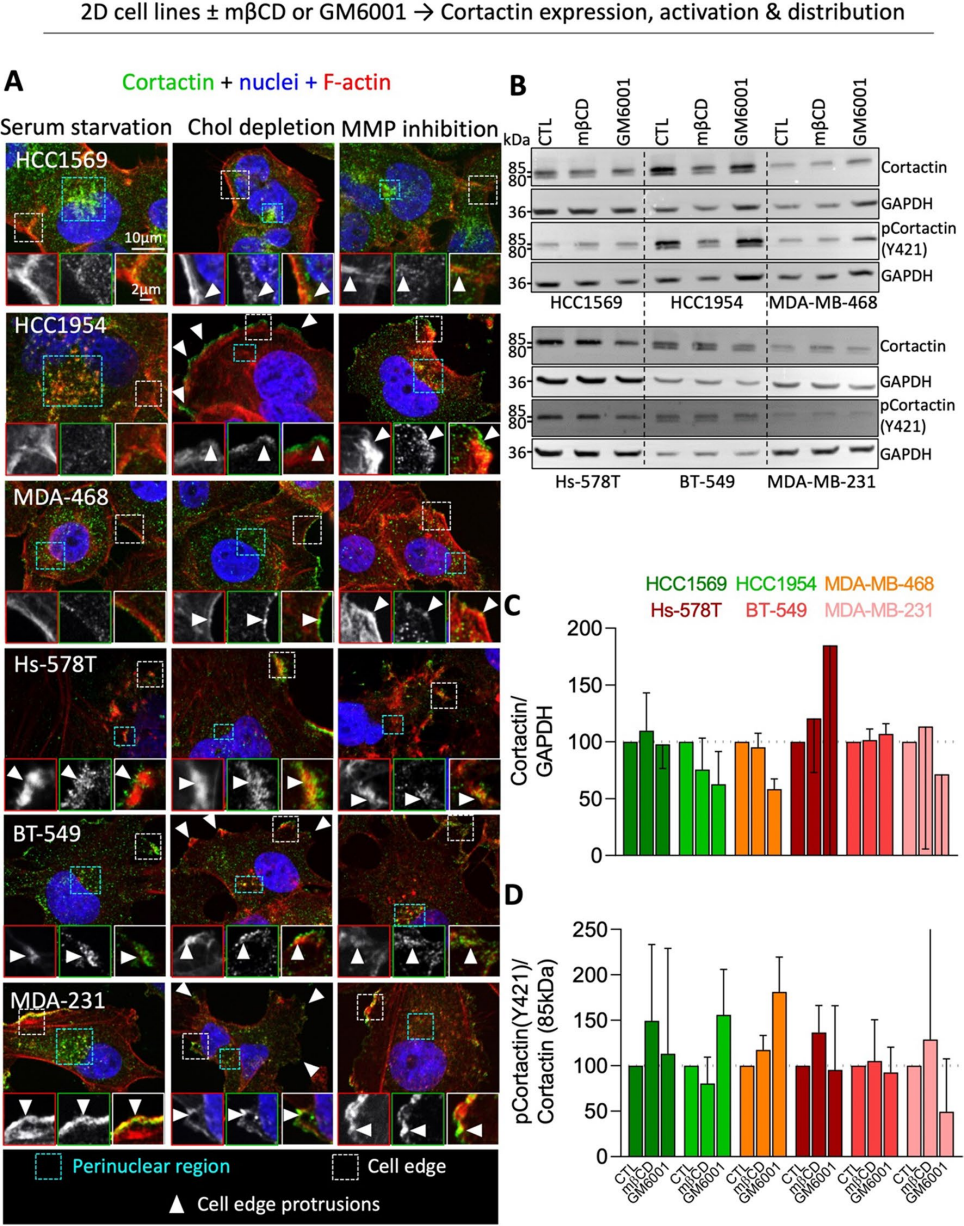
Cholesterol depletion partially delocalizes cortactin from the perinuclear region but does not affect its phosphorylation state. Cell lines were either serum-starved combined or not with 2 mM mβCD (chol depletion) for 2 h then stimulated for 4 h with serum-containing medium or serum-starved for 2 h then incubated in 10 µM GM6001-supplemented medium (MMP inhibition). All conditions were tested for cortactin distribution by confocal microscopy (**A**) and expression and activation by Western blots (**B**–**D**). **A** Confocal images of cells plated on fibronectin-coated coverslips, serum-starved or treated with mβCD or GM6001. Cells were (immuno) labeled with anti-Cortactin, Phalloidin (F-actin) and Hoechst (nuclei). Blue areas, perinuclear regions occupied by cortactin. White areas and corresponding insets, cortactin at the cell edge. White arrowheads, cell edge protrusions (n = 1–3). **B** Western blots showing the expression levels of cortactin and phosphorylated cortactin (pCortactin Y421) with both isomers at 80 kDa and 85 kDa in the 6 most invasive cell lines treated of not with mβCD or GM6001. GAPDH was used as loading control (n = 2–3). **C**, **D** Quantification of total cortactin (80 + 85 kDa forms) protein abundance (**C**) and phosphorylation level of the 85 kDa form (ratio of pCortactinY421/cortactin; **D**). Data are expressed as percentage of the untreated control (n = 2–3)

As cortactin distribution was different in the 9 cell lines, we next evaluated the potential impact of membrane chol depletion and MMP inhibition on this distribution. MβCD reduced cortactin perinuclear distribution in all cell lines although the effect was less evident in Hs-578T, potentially due to the lower cortactin perinuclear localization in these cells. GM6001 induced a smaller effect (Fig. 3A blue area size). The reduced perinuclear distribution of cortactin upon chol depletion and MMP inhibition was accompanied by an increased association with the cell edges whatever the cell line (Fig. 3A arrowheads in white insets). Moreover, the 3 gelatin-degrading cell lines (i.e. HCC1954, BT-549 and MDA-MB-231) formed small peripheral protrusions all around (Fig. 3A white arrowheads in main Figure panels). These effects of mßCD did not result from toxicity and were specific to cortactin, as the reduction of invadopodia size and number upon mβCD were completely restored upon chol repletion (Additional file 1: Fig. S4B) and other cytoskeletal components such as microtubules were unaffected by the treatment (Additional file 1: Fig. S6). A decrease of cortactin expression and activation was also ruled out (Fig. 3B–D). In conclusion, chol depletion partly delocalized cortactin distribution from the perinuclear region to cell edges, forming small non-polarized protrusions in the 3 gelatin-degrading cell lines.

### Membrane cholesterol-enriched domains are more abundant at the ECM-free side of aggressive cell lines and at the ECM-contact side of gelatin-degrading cells

**Fig. 4:**
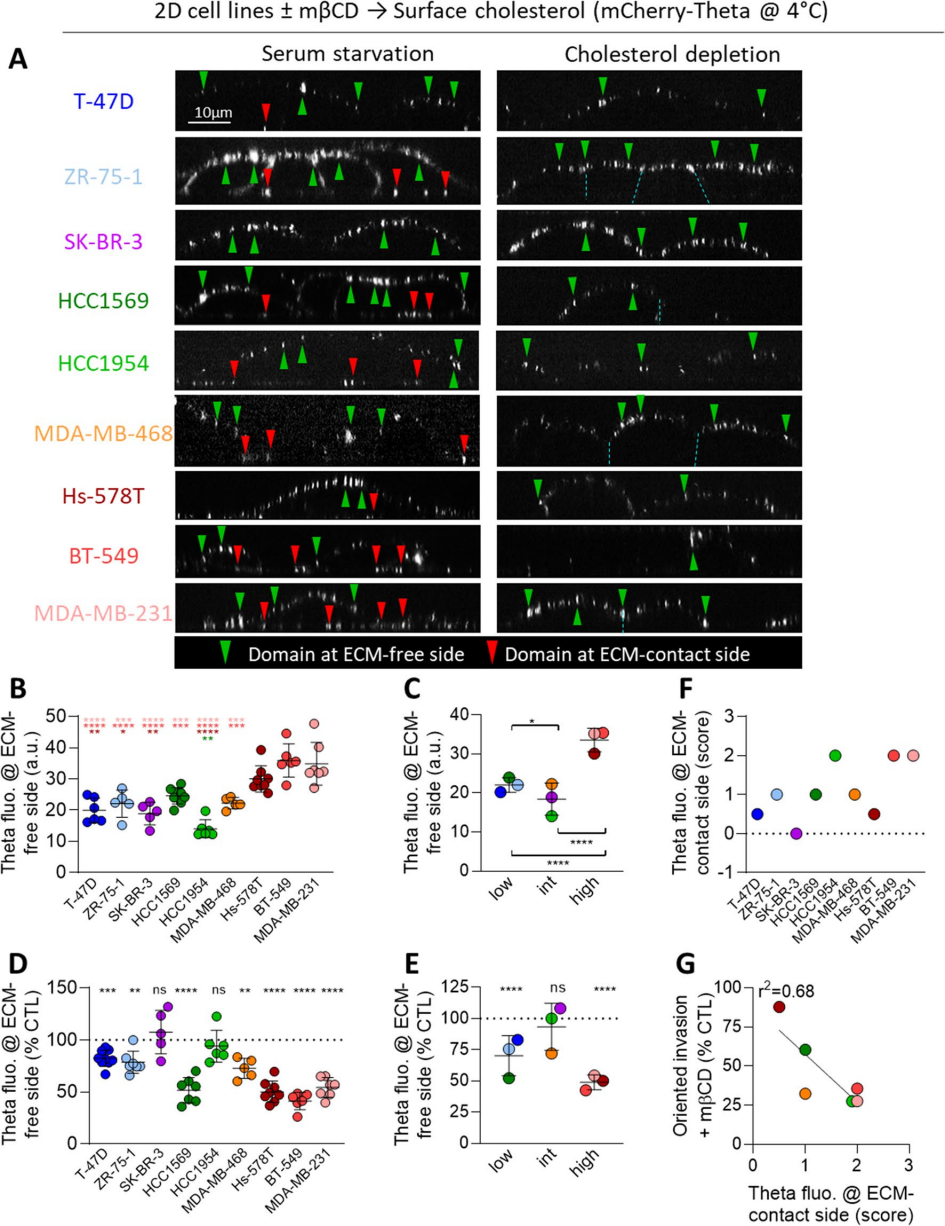
Cholesterol-enriched domains are more abundant at the ECM-contact side of gelatin-degrading cell lines. **A** X–Z reconstructions of confocal images of 9 breast cancer cell lines plated on fibronectin-coated coverslips, serum-starved combined or not with 2mM mβCD (chol depletion) for 2 h and labeled at 4 °C with the mCherry-Theta toxin fragment specific to endogenous chol. Green and red arrowheads, chol-enriched domains at ECM-free and ECM-contact cell sides respectively. **B**, **D** Quantification of the Theta ECM-free side fluorescence intensity of the 9 cell lines treated (**D**) or not (**B**) with mβCD. Each data point represents the average fluorescence intensity value of 1 coverslip (120–464 cells from n = 23–62 images from 3–5 independent experiments). **C**, **E** Theta fluorescence intensity at ECM-free side of cell lines treated (**E**) or not (**C**) with mβCD and divided in 3 groups based on aggressiveness determined in Additional file 1: Table S2: low (score ranging from 0 to 4), intermediate (int; score ranging from 5 to 8) and high aggressiveness (score ranging from 9 to 13). Each data point represents the mean fluorescence intensity value per cell line. Ordinary one-way ANOVA followed by Tukey post-hoc comparison test (**B, D**) and one sample *t* test (**C**, **E**). **F** Theta fluorescence score at the ECM-contact side attributed to the 9 cell lines based on X–Z reconstructions of Theta-labeled cells (see Additional file 1: Materials and methods section). Score ranged from 0 to 2. Higher scores were attributed to cells with higher chol-enriched domains at the ECM-contact side. **G** Inverse correlation between the Theta fluorescence score at the cell ECM-contact side and oriented invasion upon mβCD

To next determine which pool of chol could be involved in cell invasion and cortactin distribution at invadopodia in gelatin-degrading cell lines, PM chol distribution was analyzed by cell labeling at 4 °C with the chol-specific mCherry-Theta toxin fragment [24, 27]. X–Z reconstructions of confocal images revealed the presence of submicrometric domains at the ECM-free side of all cell lines (Fig. 4A green arrowheads). These domains were significantly increased in the 3 aggressive basal-like TN Hs-578T, BT-549 and MDA-MB-231 (Fig. 4B, C) but did correlate neither with cell line aggressiveness nor with the extent of cell line oriented invasion (Additional file 1: Fig. S7A, B). Upon chol depletion, the abundance of chol-enriched domains at the ECM-free side was particularly reduced in the same 3 cell lines as well as in HCC1569 (Fig. 4D) but again no relation with cell line aggressiveness or extent of oriented invasion was observed (Fig. 4E, Additional file 1: S7C, D).

In addition to the ECM-free side, chol-enriched domains could also be detected at the ECM-contact side of almost all cell lines. However, they were particularly abundant in the 3 gelatin-degrading cell lines (Fig. 4A, F; red arrowheads), consolidating our hypothesis that membrane chol could be involved in invadopodia formation and/or maintenance. Two additional observations supported this possibility. First, chol-enriched domains disappeared from the ECM-contact side upon chol depletion (Fig. 4A right) but not upon MMP inhibition (Additional file 1: Fig. S8). Second, the strongest decrease of oriented invasion upon chol depletion was observed in cell lines showing the highest proportion of domains at the ECM-contact side (Fig. 4G).

In conclusion, the proportion of chol-enriched domains at the ECM-contact side could help predicting breast cancer sensitivity to chol depletion while chol-enriched domains at the ECM-free side were more abundant in aggressive cell lines but only poorly correlated with cell aggressiveness.

### Cholesterol depletion affects intracellular neutral lipid partitioning in endoplasmic reticulum *vs.* lipid droplets

**Fig. 5:**
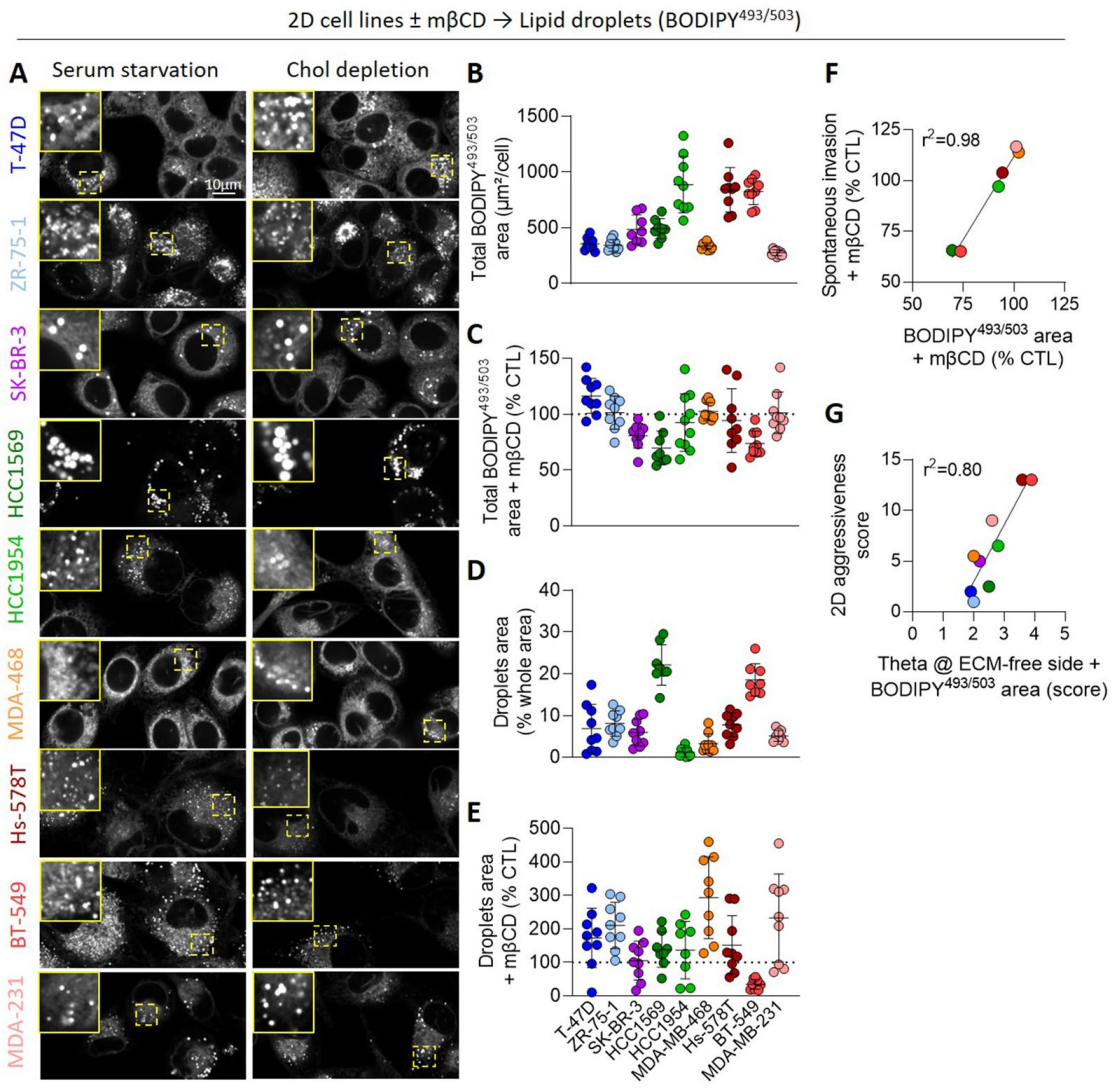
Cholesterol depletion decreases neutral lipid cell area occupancy in cell lines impaired for spontaneous invasion. The 9 cell lines were plated on fibronectin-coated coverslips, serum-starved combined or not with 2mM mβCD (chol depletion) for 2 h, labeled at 37 °C with the BODIPY^493/503^ specific to lipid droplets and then visualized by confocal microscopy. **A** Representative confocal images and insets to better evidence lipid droplet size and abundance (n = 2–3). **B**–**E** Quantification of total BODIPY^493/503^ fluorescence area per cell (**B**, **C**) and lipid droplets area occupancy (**D**, **E**) of the 9 breast cancer cell lines in untreated conditions (**B**, **D**) or upon mβCD (**C**, **E**). Each data point represents the mean value of 1 image. Graphs are representative of 1 experiment among 2–3. **F, G** Linear correlations between the total BODIPY^493/503^ fluorescence area and spontaneous invasion of cells treated with mβCD (**F**) and between Theta at cell ECM-free side combined with BODIPY^493/503^ fluorescence area score and 2D aggressiveness score (**G**). 2D aggressiveness scores are from Additional file 1: Table S2. Theta at cell ECM-free side scores were based on chol labeling with Theta toxin (Fig. 4B): higher score was attributed to cells with higher Theta fluorescence intensity at the cell ECM-free side. BODIPY^493/503^ fluorescence area scores were based on lipid droplet labeling with BODIPY^493/503^ (**B**): higher score was attributed to cells with higher total BODIPY^493/503^ fluorescence area. Each of these two later parameters were associated to a score ranging from 0 to 2, and scores were then added

To understand the lack of correlation, cell lines were compared for the amounts of intracellular neutral lipids, i.e. chol esters and triacylglycerol, through labeling with BODIPY^493/503^. Confocal vital imaging revealed differential BODIPY distribution and amounts in perinuclear dots and endoplasmic reticulum (Fig. 5A). Quantification of total BODIPY^493/503^ fluorescence area per cell indicated a stronger signal in HCC1954, Hs-578T and BT-549, a medium signal in SK-BR-3 and HCC1569, and a lower signal in T-47D, ZR-75-1, MDA-MB-468 and MDA-MB-231 (Fig. 5B). Chol depletion particularly affected HCC1569 and BT-549 (Fig. 5C). Lipid droplet specific area occupancy was the highest in the two latter cell lines and showed a tendency to increase upon chol depletion in all cell lines except in BT-549 (Fig. 5D, E), suggesting that chol depletion affected the intracellular neutral lipid partitioning in endoplasmic reticulum *vs.* lipid droplets. As HCC1569 and BT-549 were the only cell lines showing a decreased spontaneous invasion upon mβCD, this partitioning could be important for cell invasion. In agreement with this observation, spontaneous invasion upon mβCD positively correlated with the extent of neutral lipids upon chol depletion (Fig. 5F). Moreover, when considering both the chol-enriched domains at the ECM-free side and the intracellular neutral lipid content, a positive correlation appeared with aggressiveness (Fig. 5G), which was not the case when considering only the chol-enriched domains (Fig. S7A) or the intracellular neutral lipid content (data not shown).

### Cholesterol depletion but not MMP inhibition impairs breast cancer cell line spheroid invasion whatever the subtype and mechanism of invasion

**Fig. 6:**
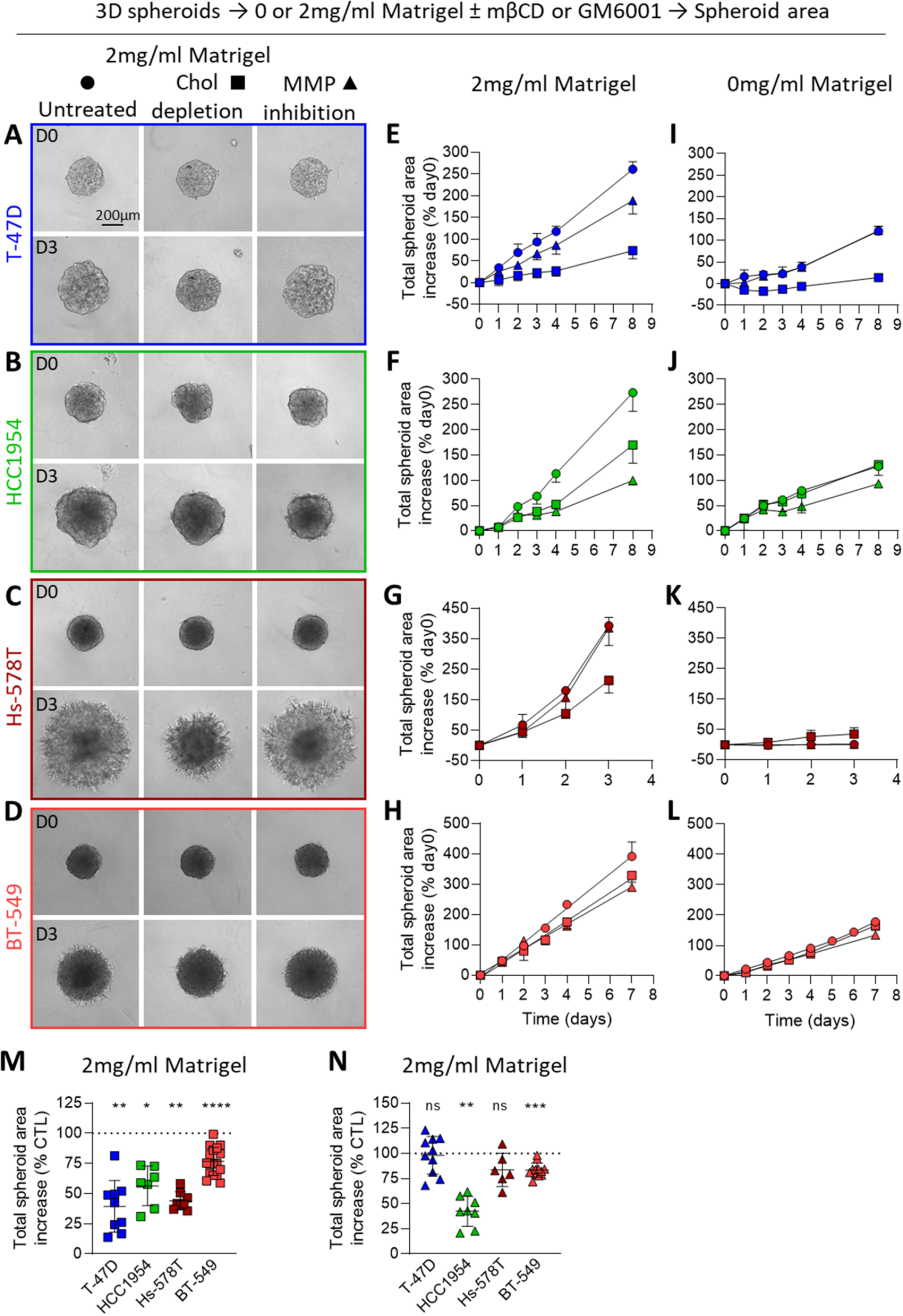
Cholesterol depletion but not MMP inhibition reduces the size of the four breast cancer spheroids. T-47D, HCC1954, Hs-578T and BT-549 spheroids were grown for 3 days in ultra-low-attachment plates, then embedded (**A**–**D**, **E**–**H**, **M**, **N**) or not (**I**–**L**) in 2 mg/mL Matrigel supplemented or not with 1.25 mM mβCD (chol depletion) or 30 µM GM6001 (MMP inhibition) and allowed to grow for 8 days. **A**–**D** Representative phase contrast images of spheroids treated or not with mβCD or GM6001 at days 0 (D0) and 3 (D3). **E**–**L** Time-course quantification of total spheroid area increase of the four spheroids embedded (**E**–**H**) or not (**I**–**L**) in Matrigel and treated or not (circles) with mβCD (squares) or GM6001 (triangles). Data are expressed as percent increase of day 0. Graphs are representative of 1 experiment with 3–5 spheroids per condition (in total 5–18 spheroids from 2–4 independent experiments). **M**, **N** Quantification of total spheroid area increase upon chol depletion (**M**) or MMP inhibition (**N**) at day 3. Data are expressed as percent increase of day 3 untreated condition. Each data point represents 1 spheroid (in total 6–17 spheroids from 2–5 independent experiments). Wilcoxon signed-rank test

We finally asked whether the above observations in 2D could be extended to 3D spheroids and if the effect of chol depletion on the insensitive Hs-578T could be revealed in 3D. Spheroids were generated from T-47D, HCC1954, Hs-578T and BT-549 cells, chosen for their ability to form round spheroids and their molecular subtypes (luminal A-like, HER2-enriched and basal-like TN; Additional file 1: Table S1; Additional file 1: Fig. S9A). All 4 spheroids invaded the Matrigel whatever the Matrigel concentration (Fig. 6A–D, Additional file 1: Fig. S9B). Furthermore, contrarily to our previous findings in 2D invasion assay, Hs-578T spheroids were drastically more invasive in 3D than BT-549 spheroids (Fig. 6C, D, G, H).

**Fig. 7:**
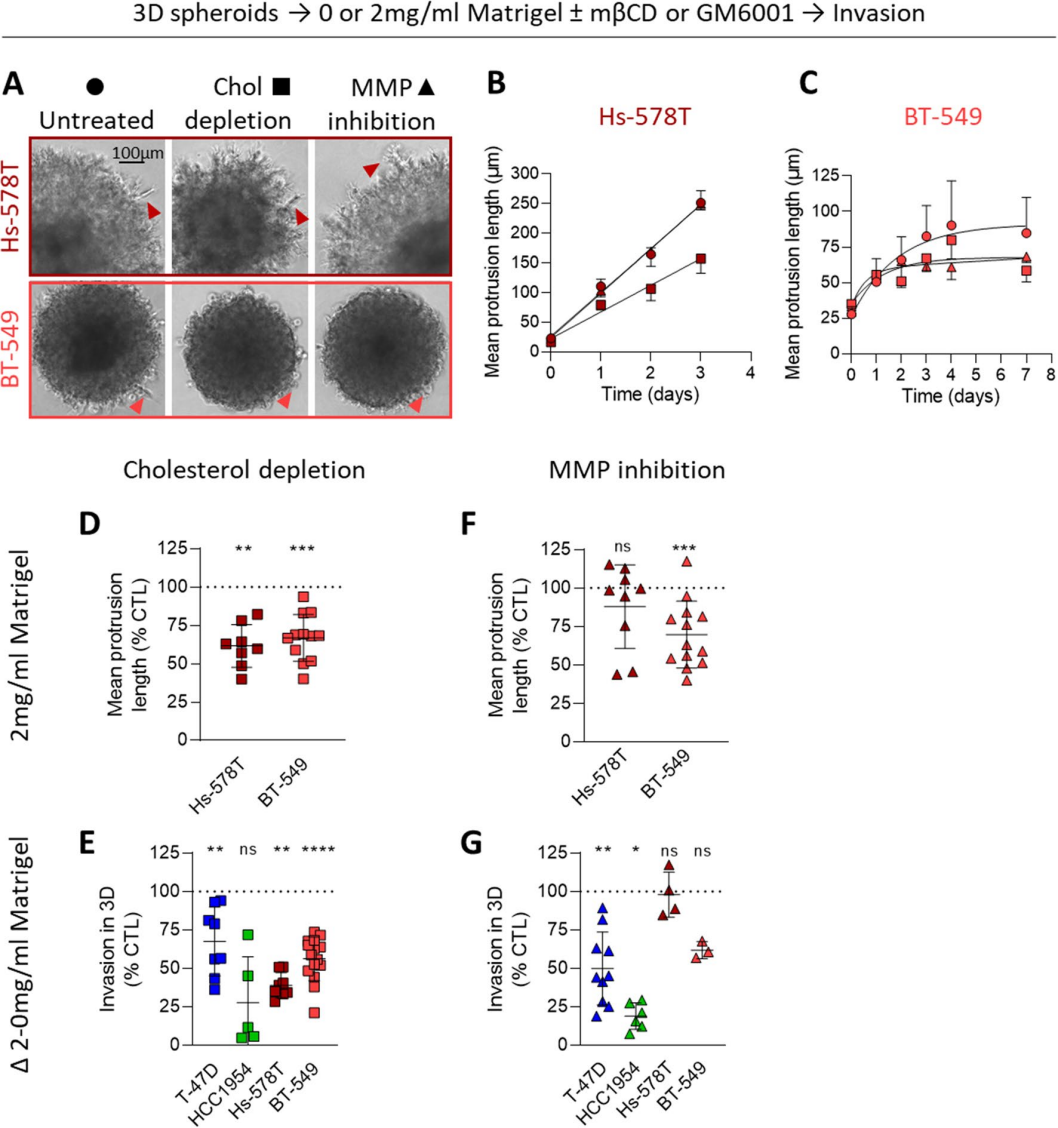
Cholesterol depletion but not MMP inhibition reduces the 3D invasion of the four breast cancer spheroids. T-47D, HCC1954, Hs-578T and BT-549 spheroids were grown and treated exactly as in Fig. 6 and then tested for protrusion length and 3D invasion into 2 mg/mL Matrigel. **A** Representative phase contrast images at day 3 of invading Hs-578T and BT-549 spheroids, treated or not with mβCD (chol depletion) or GM6001 (MMP inhibition). Arrowheads, spheroid protrusions. **B**, **C** Time-course quantification of the mean protrusion length of Hs-578T (**B**) and BT-549 (**C**) spheroids embedded in Matrigel and treated or not (circles) with mβCD (squares) or GM6001 (triangles). Graphs are representative of 1 experiment with 5 spheroids per condition (in total 8–18 spheroids from 2–4 independent experiments). **D**–**G** Quantification of mean protrusion length at day 3 (**D**, **F**) and invasion in 3D at the last day of analysis (**E**, **G**) of cell lines treated with mβCD or GM6001. 3D invasion potential in E, G was obtained by subtracting the spheroid area increase without Matrigel (Fig. 6I-L) from the area increase with Matrigel (Fig. 6E-H). Each data point represents 1 spheroid (in total 3–15 spheroids from 2–5 independent experiments). Wilcoxon signed-rank test

To test the potential effect of chol depletion in 3D, spheroids were treated with 1.25 mM mβCD and spheroid growth in Matrigel was measured over a week. Strikingly, chol depletion seemed to reduce the spheroid area increase of all 4 cell lines whereas MMP inhibition specifically reduced the spheroid area increase of HCC1954 and BT-549 but not T-47D and Hs-578T (Fig. 6E–H, M, N, Additional file 1: Fig. S10). This suggested a different mode of invasion in T-47D and Hs-578T cell lines, which did not involve MMP activity but required adequate membrane chol content, contrarily to HCC1954 and BT-549 which depended on both. Moreover, the mβCD-induced effect observed in T-47D spheroids could partly result from cell proliferation impairment, as suggested by the absence of spheroid growth without Matrigel (Fig. 6I–L, Additional file 1: Fig. S10). Therefore, to assess the effect of chol depletion specifically on cell invasion and not proliferation, we also measured spheroid protrusion length and 3D invasion potential by subtracting the area increase without Matrigel (Fig. 6I–L) from the area increase with Matrigel (Fig. 6E–H). Both mean protrusion length and 3D invasion potential were impaired by mβCD in all cell line spheroids, contrarily to GM6001 which only reduced spheroid protrusion length of BT-549 and 3D invasion potential of HCC1954 and BT-549 (Fig. 7). Thus, chol depletion similarly impaired invasion in all spheroids tested. Surprisingly, the effect of mßCD on spheroid size and invasion seemed less pronounced in the BT-549 cell line which shows the highest PM chol-enriched domains in 2D. This could be due to the higher compaction of these spheroids (Fig. 6A–D), allowing for lower penetration of mβCD. In agreement with this hypothesis, increasing mβCD concentration in the BT-549 spheroids produced a concentration-dependent decrease of spheroid invasion, while growth remained unaffected at least until day 4 (Additional file 1: Fig. S11A–C). In contrast, increasing GM6001 concentration did not produce a stronger effect (Additional file 1: Fig. S11D–F).

Our results showed for the first time the contribution and the possibility to target PM chol to reduce breast cancer cell invasion using spheroid models while MMP inhibition was less powerful.

## Discussion

### Main observations

Using a panel of 9 breast cancer cell lines grouped in 4 subtypes matching breast tumor classification, we showed that PM chol was required for the 2D invasion of the 6 invasive cell lines, whatever the subtype, mutations, extent of invasion or aggressiveness. Nevertheless, the effect of chol depletion was more important in cell lines able to degrade gelatin and considerably lower in the TN Hs-578T. Such differential effects disappeared in 3D spheroids. In both 2D and 3D, targeting PM chol was more powerful than MMP inhibition to reduce breast cancer invasion. We also found that chol depletion impaired cortactin distribution in the perinuclear region in the 6 invasive cell lines. All cell lines exhibited PM chol-enriched submicrometric domains which were more abundant at the ECM-free side of aggressive cells and at the ECM-contact side of gelatin-degrading cells. In addition to the PM chol content, intracellular lipid droplets seemed important for cell aggressiveness and invasiveness. Our data open the way to use surface chol combined with lipid droplet labeling as breast cancer aggressiveness marker and to test other chol-targeting drugs in more complex models to further evaluate whether this lipid could represent a potential target in breast cancer therapy.

### Cholesterol depletion allows to reduce breast cancer cell invasion on 2D cell lines and 3D spheroids

Conflicting data regarding the role of membrane chol in breast cancer cell invasion exist in the literature. Indeed, lower PM chol contents were shown to promote breast cancer cell migration by increasing membrane fluidity and deformability [20, 21], while conversely, PM chol depletion was reported to reduce TNBC cell line migration and invasion of malignant MCF10CAIa and MDA-MB-231 cell lines [13, 18, 19]. As the different subtypes of breast cancer cell lines exhibit different invasion capacity and aggressiveness [28], these conflicting data suggested that PM chol could differentially contribute to cell invasion. By comparing the 2D invasion potential of 9 breast cancer cell lines representing 4 molecular subtypes, we here demonstrated that chol depletion reduced the oriented invasion of all invasive cell lines, albeit to varying degrees, which was neither related to their subtype nor aggressiveness but appeared more important in the 3 gelatin-degrading cell lines and considerably lower in Hs-578T. These results obtained in 2D contrasted with those observed in 3D spheroids. First, all spheroids, including the luminal-like T-47D cell line that was non-invasive in 2D, were able to invade the Matrigel whatever their subtype. Second, chol depletion impaired all spheroid 3D invasion potential, including Hs-578T, irrespective of their subtype or aggressiveness. The different results observed on the same cell lines in 2D and 3D could result from their differential complexity. 3D spheroids better mimic tumor features than 2D cultures in terms of gene expression, physico-chemical gradients and cell–cell and cell-ECM interactions [29]. Moreover, growing cells in 2D offers unlimited access to nutrients, oxygen, growth factors and metabolites, contrarily to 3D spheroids in which diffusion is limited to the outer cell layers, forming a gradient with the central core [30]. Chol metabolism is also differentially regulated when switching from 2D to 3D culture as attested by the differential expression of chol biosynthesis genes and effect of chol synthesis inhibitors, like statins, on breast cancer cell growth and invasion [9, 11, 31].

### The effect of cholesterol depletion on Hs-578T oriented invasion is very low in 2D but drastically enhanced in 3D

Unexpectedly, Hs-578T cells showed drastic differences of invasion impairment in response to chol depletion in 2D and 3D models which could be explained by the following possibilities. First, Hs-578T cells cultured in 2D are flat and isolated, whereas in 3D culture, their natural cellular structures with high compaction and cell contacts are preserved [29]. In 2D, the formation of tight junctions was shown to depend on PM chol content [32]. Second, 3D cultures also direct cells to modify the organization of surface receptors to sustain the increased dynamic of cell-ECM interactions that are absent in 2D [29]. Third, Hs-578T are known to secrete hyaluronan which promotes increased cell-ECM binding via CD44 and invasion [33]. This secretion could be higher in 3D than 2D.

### Cholesterol depletion impairs cortactin subcellular distribution

Cortactin was expressed and activated similarly in all 9 cell lines, but its differential distribution appeared to depend on PM chol, potentially modulating its binding to partners or post-translational modifications. In fact, cortactin comprises a (i) N-terminal acidic domain that interacts with the Arp2/3 actin nucleator complex, (ii) an actin filament-binding region comprising 6.5 tandem repeats, (iii) a proline-rich domain prone to post-translational regulation and (iv) a C-terminal Src homology 3 (SH3) domain that interacts with other proteins [34]. Cortactin localization is known to be mainly influenced by its binding to F-actin present in specific cellular regions including lamellipodia, invadopodia, late endosome and Golgi, and to the Arp2/3 complex [34]. Moreover, deacetylation of F-actin-binding repeats promotes cortactin translocation to leading edges and cell motility [35]. Cortactin translocation from the cytoplasm to the cell periphery is also mediated by the activation of the small GTPase Rac1 [36] which localizes to PM rafts and depends on PM chol for its activation [37, 38]. In addition, cortactin binding to other partners via its SH3 domain and its multiple post-translational modifications including phosphorylation could play a role in its localization in invadopodia and depend on membrane chol. Indeed, cortactin binds to N-WASP, an actin nucleation promoting factor that localizes in rafts at invadopodia [39]. Furthermore, cortactin is phosphorylated by the tyrosine kinases Src and Arg which both localize to invadopodia of breast cancer cell lines [40], the former being localized to rafts [41].

### Surface cholesterol combined with lipid droplet labeling as marker of breast cancer cell aggressiveness in 2D

Currently, the determination of breast tumor aggressiveness relies on the assessment of the stage and molecular subtype which influence the prognosis, risks of recurrence and mortality, and treatment options [42]. Nonetheless, up to 20% of breast cancer cases, especially the aggressive TNBC subtype, will relapse and potentially evolve in metastatic disease and death [43]. This could partly be explained by the large degree of molecular heterogeneity that remains unappreciated across and within each subtype despite the development of multigene expression assays [44]. Thus, identifying new biomarkers could enable better determination of breast tumor aggressiveness. We here proposed the use of surface chol content. While significantly higher at the surface of the most aggressive TN cell lines in 2D cultures, this parameter alone was not able to distinguish cell lines of low and intermediate aggressiveness. This could be due to the lack of consideration of ECM-contact side and intracellular chol pools. Indeed, chol present at the ECM-contact side of the 3 gelatin-degrading cell lines participated in invadopodia formation and/or function, a parameter we considered when assessing cell aggressiveness. Moreover, both PM chol contents were probably directly connected, as suggested by our previous work that reported that the ECM-contact side PM chol found next to invadopodia arises from the ECM-free side by endocytosis [13]. Hence, when considering both chol-enriched domains at ECM-free side and intracellular neutral lipid contents, a positive correlation appeared with cell aggressiveness. The association of intracellular chol content with breast cancer aggressiveness is not new. For instance, increased cholesteryl ester levels have been observed in tumors with higher grade, Ki-67 and necrosis, as well as in HER2-enriched and TNBC tumors [45]. Altogether, our data revealed for the first time that co-labeling both PM chol content with the Theta toxin fragment and lipid droplets with BODIPY^493/503^ could offer predictive insight on breast cancer cell aggressiveness.

### Surface cholesterol as a therapeutic target in breast cancer?

In the past, broad-spectrum MMP inhibitors failed in early clinical trials mainly due to lack of inhibitor specificity [22, 23]. Our results suggest that partial depletion of PM chol was more effective than global MMP inhibition to limit breast cancer cell invasion. First, mβCD targeted all cell lines whatever their subtype and dependence to MMPs for invasion. Second, in the cell lines endowed with MMP-mediated gelatin-degrading activity, the extent of invasion decrease was more important upon mβCD than GM6001. Third, unlike MMP inhibition, chol depletion reduced the 3D invasion of all 4 tested breast cancer cell lines. Nevertheless, using membrane chol-targeting agents to minimize cancer cell invasion would not only require many adjustments including improving drug diffusion within the tumor but also testing other chol-targeting molecules in more complex breast cancer research models, including xenografts in nude mice. Moreover, additional research is needed to evaluate the potential synergistic effects with existing therapies or newly-developed strategies. In this context, exploring integrins as anticancer strategies in metastatic breast cancer has gained attention during last years. For instance, a very recent study revealed the anti-angiogenic activity of a disintegrin-like protein derived from snake venom, leading to inhibition of tumor growth and metastasis and providing insights into the development of anti-tumor and anti-angiogenic strategies to improve TNBC treatments [46].

## Conclusions

Our study revealed the implication of PM chol in the invasion of the six invasive cell lines, no matter their subtype or aggressiveness. Nevertheless, the effect of chol depletion was stronger in the three cell lines able to degrade gelatin. Spheroid invasion was also reduced after chol depletion in all breast cancer subtypes tested. Notably, targeting chol was more powerful than MMP inhibition in reducing cell invasion in 2D and 3D culture models. Our work opens the way to further investigate the potential use of PM chol as anticancer therapeutic target using other chol-targeting agents and in vivo models.

### Supplementary Information

**Additional file 1.** **Supplementary Materials and Methods.** Cell cholesterol repletion and 2D cell morphology. **Table S1**. Overview of subtype, tumor origin, molecular markers information and mutations of the 9 breast cancer cell lines used in this study. **Table S2**. Aggressiveness score in 2D of the 9 breast cancer cell lines. **Figure S1**. Characterization of the 9 breast cancer cell lines for molecular markers. **Figure S2**. Cholesterol depletion does not affect 2D morphology of the 9 breast cancer cell lines. **Figure S3**. The 9 cell lines differ by the extent of invasion and cholesterol depletion but not MMP inhibition reduces spontaneous invasion in 2D of 2 cell lines. **Figure S4**. Cholesterol depletion only slightly decreases Hs-578T invasion in 2D and reversibly affects invadopodia formation in MDA-MB-231, as shown by cholesterol repletion. **Figure S5**. Only three cell lines are able to degrade the gelatin. **Figure S6**. Cholesterol depletion does not affect the distribution of microtubules in Hs-578T and BT-549 cell lines. **Figure S7**. Cholesterol-enriched domains at ECM-free side do correlate neither with breast cancer cell aggressiveness nor with invasion potential. **Figure S8**. Cholesterol depletion, but not MMP inhibition, impairs cholesterol-enriched domains at the ECM-contact side in ZR-75-1 and BT-549 cells. **Figure S9**. Differential breast cancer cell line spheroid shape and 3D invasion in Matrigel. **Figure S10**. Cholesterol depletion reduces spheroid invasion but not growth. **Figure S11**. Cholesterol depletion, but not MMP inhibition, induces a dose-dependent decrease of BT-549 spheroid invasion.

## Data Availability

The original contributions presented in the study are included in the article Supplementary Material and Supplementary figures. The datasets used and/or analyzed during the current study are available from the corresponding author upon reasonable request.

## Abbreviations

chol: Cholesterol
ECM: Extracellular matrix
MMP: Matrix metalloproteinase
mβCD: Methyl-β-cyclodextrin
PM: Plasma membrane
TN: Triple negative
TNBC: Triple negative breast cancer
