# Supplementary material for: Targeting cholesterol impairs cell invasion of all breast cancer types
Source: Cancer Cell Int. 2024 Jan 10;24:27. doi: 10.1186/s12935-023-03206-z (PMC10782689; doi:10.1186/s12935-023-03206-z)
Supplement: Supplementary file 1 — Additional file 1. Supplementary Materials and Methods. Cell cholesterol repletion and 2D cell morphology. Table S1. Overview of subtype, tumor origin, molecular markers information and mutations of the 9 breast cancer cell lines used in this study. Table S2. Aggressiveness score in 2D of the 9 breast cancer cell lines. Figure S1. Characterization of the 9 breast cancer cell lines for molecular markers. Figure S2. Cholesterol depletion does not affect 2D morphology of the 9 breast cancer cell lines. Figure S3. The 9 cell lines differ by the extent of invasion and cholesterol depletion but not MMP inhibition reduces spontaneous invasion in 2D of 2 cell lines. Figure S4. Cholesterol depletion only slightly decreases Hs-578T invasion in 2D and reversibly affects invadopodia formation in MDA-MB-231, as shown by cholesterol repletion. Figure S5. Only three cell lines are able to degrade the gelatin. Figure S6. Cholesterol depletion does not affect the distribution of microtubules in Hs-578T and BT-549 cell lines. Figure S7. Cholesterol-enriched domains at ECM-free side do correlate neither with breast cancer cell aggressiveness nor with invasion potential. Figure S8. Cholesterol depletion, but not MMP inhibition, impairs cholesterol-enriched domains at the ECM-contact side in ZR-75-1 and BT-549 cells. Figure S9. Differential breast cancer cell line spheroid shape and 3D invasion in Matrigel. Figure S10. Cholesterol depletion reduces spheroid invasion but not growth. Figure S11. Cholesterol depletion, but not MMP inhibition, induces a dose-dependent decrease of BT-549 spheroid invasion. [file 12935_2023_3206_MOESM1_ESM.docx]

**Supplementary data**

**Materials and Methods**

**Cell cholesterol repletion.** Chol-depleted cells were subsequently incubated in serum-free medium containing mβCD charged with chol (Sigma-Aldrich #C4951) in a 6:1 molar ratio for 1 h.

**2D cell morphology.** Cells were seeded on fibronectin-precoated coverslips, starved or treated for 2 h with mβCD, then observed with a wide-field fluorescence microscope Observer Z1 (20x objective).

**
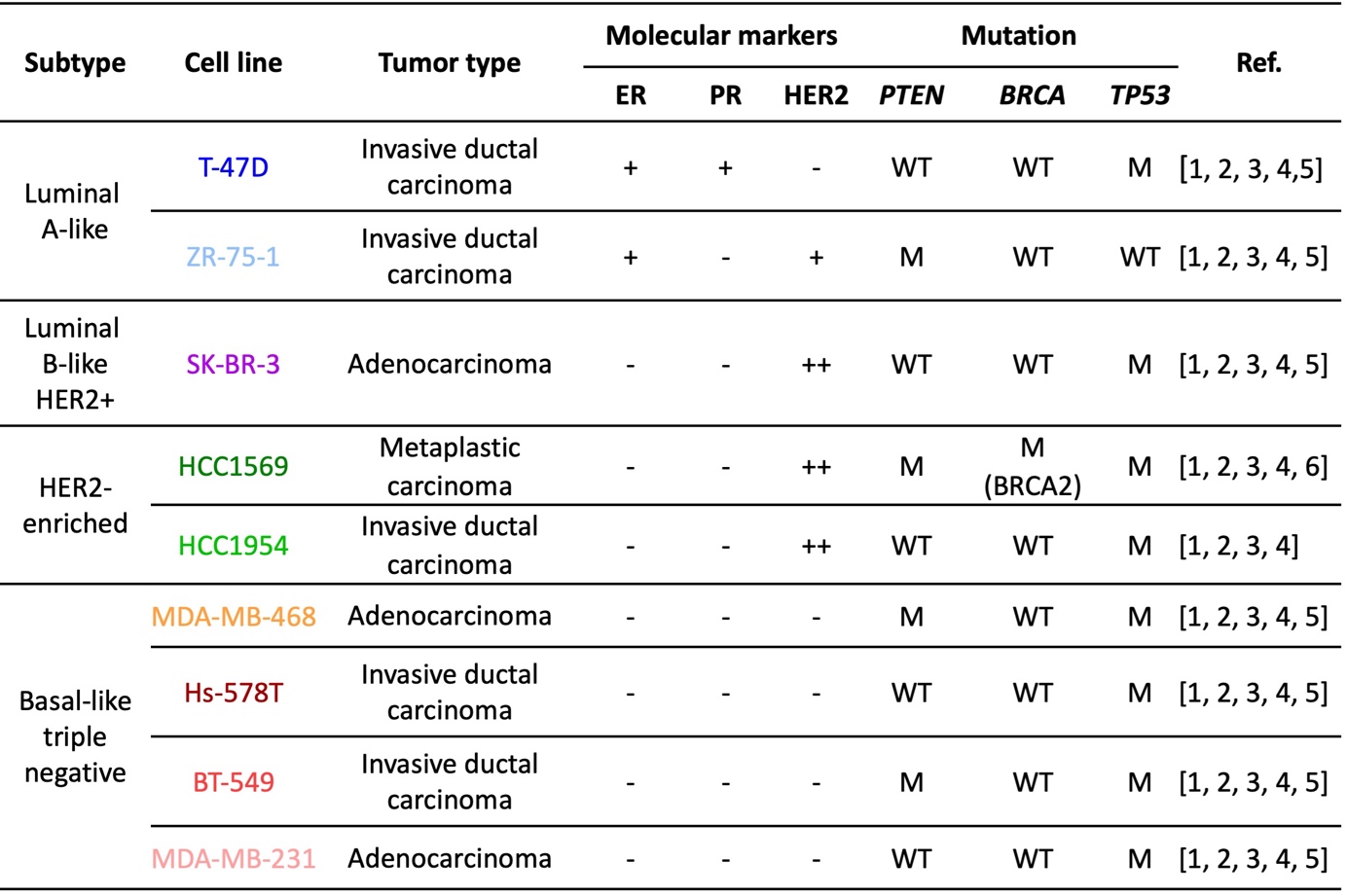
Table S1. Overview of subtype, tumor origin, molecular markers information and mutations of the 9 breast cancer cell lines used in this study.** Each breast cancer cell line was attributed a color code and categorized into 4 subtypes: luminal A-like, luminal B-like HER2 positive (HER2+), HER2-enriched and basal-like triple negative. Subtyping was established according to references listed in the column ‘References’ and the protein expression of the 3 molecular markers (ER, estrogen receptor; PR, progesterone receptor; HER2, human epidermal growth factor receptor 2; Fig. S1A-C). For protein expression: +, expression; ++, overexpression; -, no expression. The ‘Tumor type’ column shows the histopathology of the tumor where the cell lines were originally derived. The ‘Mutation’ column shows the mutational status of 3 commonly mutated genes in breast cancer (*PTEN*, phosphatase and tensin homolog; *BRCA*, breast cancer gene; *TP53*, tumor protein 53). For gene mutation: M, mutation; WT, wild-type gene.

1. Dai, X., et al., Breast Cancer Cell Line Classification and Its Relevance with Breast Tumor Subtyping. J Cancer, 2017. 8(16): p. 3131-3141.

2. Kao, J., et al., Molecular profiling of breast cancer cell lines defines relevant tumor models and provides a resource for cancer gene discovery. PLoS One, 2009. 4(7): p. e6146.

3. Neve, R.M., et al., A collection of breast cancer cell lines for the study of functionally distinct cancer subtypes. Cancer Cell, 2006. 10(6): p. 515-27.

4. Riaz, M., et al., miRNA expression profiling of 51 human breast cancer cell lines reveals subtype and driver mutation-specific miRNAs. Breast Cancer Res, 2013. 15(2): p. R33.

5. Hollestelle, A., et al., Distinct gene mutation profiles among luminal-type and basal-type breast cancer cell lines. Breast Cancer Res Treat, 2010. 121(1): p. 53-64.

6. Weigelt, B., P.H. Warne, and J. Downward, PIK3CA mutation, but not PTEN loss of function, determines the sensitivity of breast cancer cells to mTOR inhibitory drugs. Oncogene, 2011. 30(29): p. 3222-33.

**
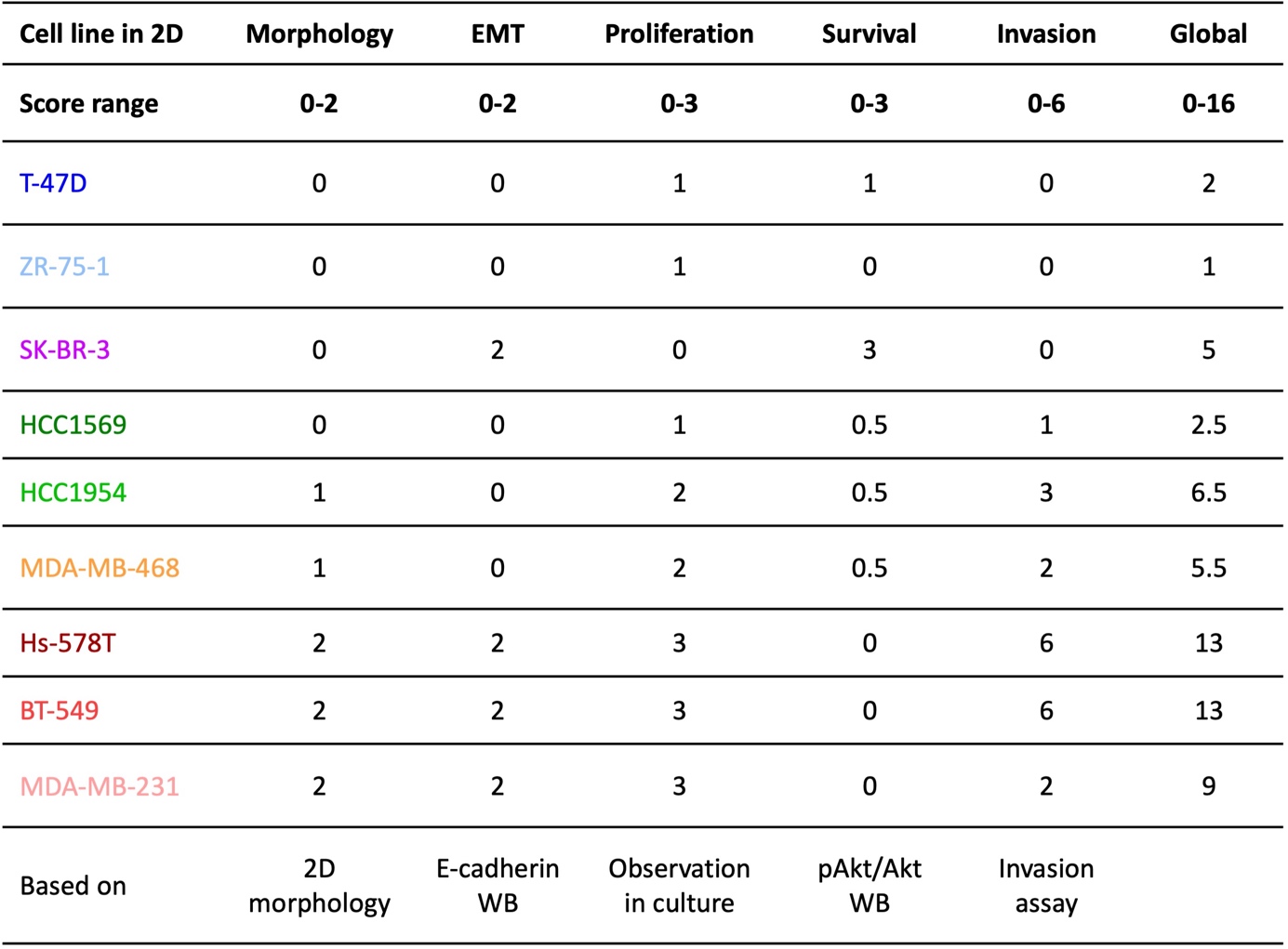
Table S2. Aggressiveness score in 2D of the 9 breast cancer cell lines.** The aggressiveness score in 2D was determined based on cell morphology, epithelial-mesenchymal transition (EMT), proliferation, survival and invasion potential. Cell morphology score was assessed by observation of 2D morphology in culture (Fig. S2): lower score was attributed to clustered cells with high cell-cell adhesions, and higher score to isolated stellate cells. EMT score was based on expression level of E-cadherin: higher score was attributed to cells downregulating E-cadherin (Fig. S1D). Proliferation score was assessed by observation made in 2D culture: higher score was attributed to cells with higher proliferation rate. Survival was based on the phosphorylated-Akt/Akt ratio, an indicator of the PI3K/Akt survival pathway activation (Fig. S1E,F): higher score was attributed to cells with higher ratio. Invasion potential was assessed by 2D Matrigel invasion assay (Fig. S3A-C): higher score was attributed to cells with higher mean number of invading cells per field. The different scores corresponding to the different parameters were then added to determine the 2D aggressiveness score for each cell line. The higher the score, the more aggressive the breast cancer cell line in 2D, the maximal possible score being 16.

**
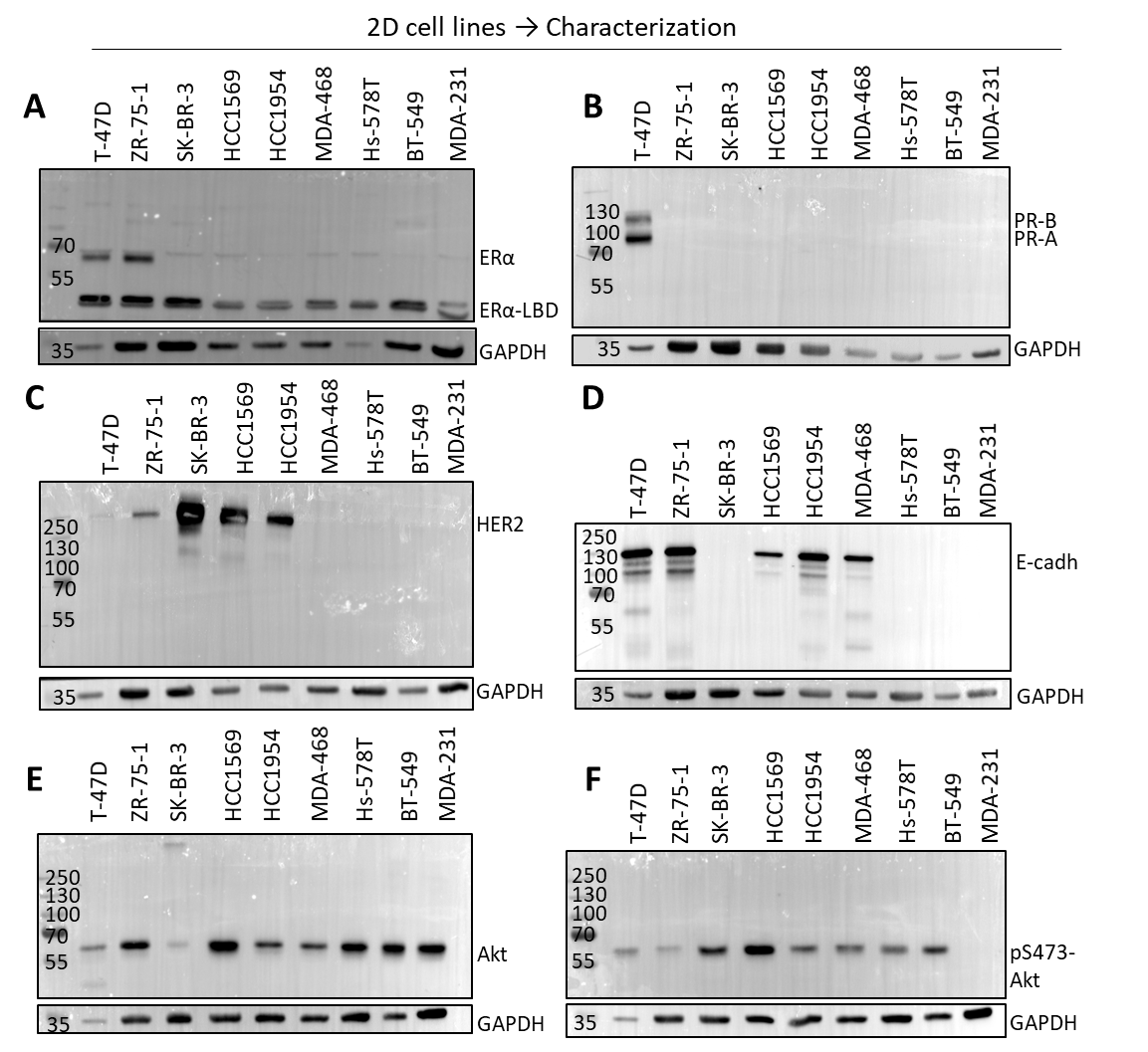
Figure S1. Characterization of the 9 breast cancer cell lines for molecular markers.** Western blots showing the expression level of estrogen receptor alpha (ERα; **A**), isoforms A and B of progesterone receptor (PR-A and PR-B; **B**), human epidermal growth factor receptor-2 (HER2; **C**), E-cadherin (E-cadh; **D**), Akt (**E**) and phosphorylated-Akt at serine 473 (pS473-Akt; **F**) of the 9 breast cancer cell lines. ERα-LBD, ligand binding domain of ER alpha. GAPDH was used as loading control (n=2-3 for each protein).

**
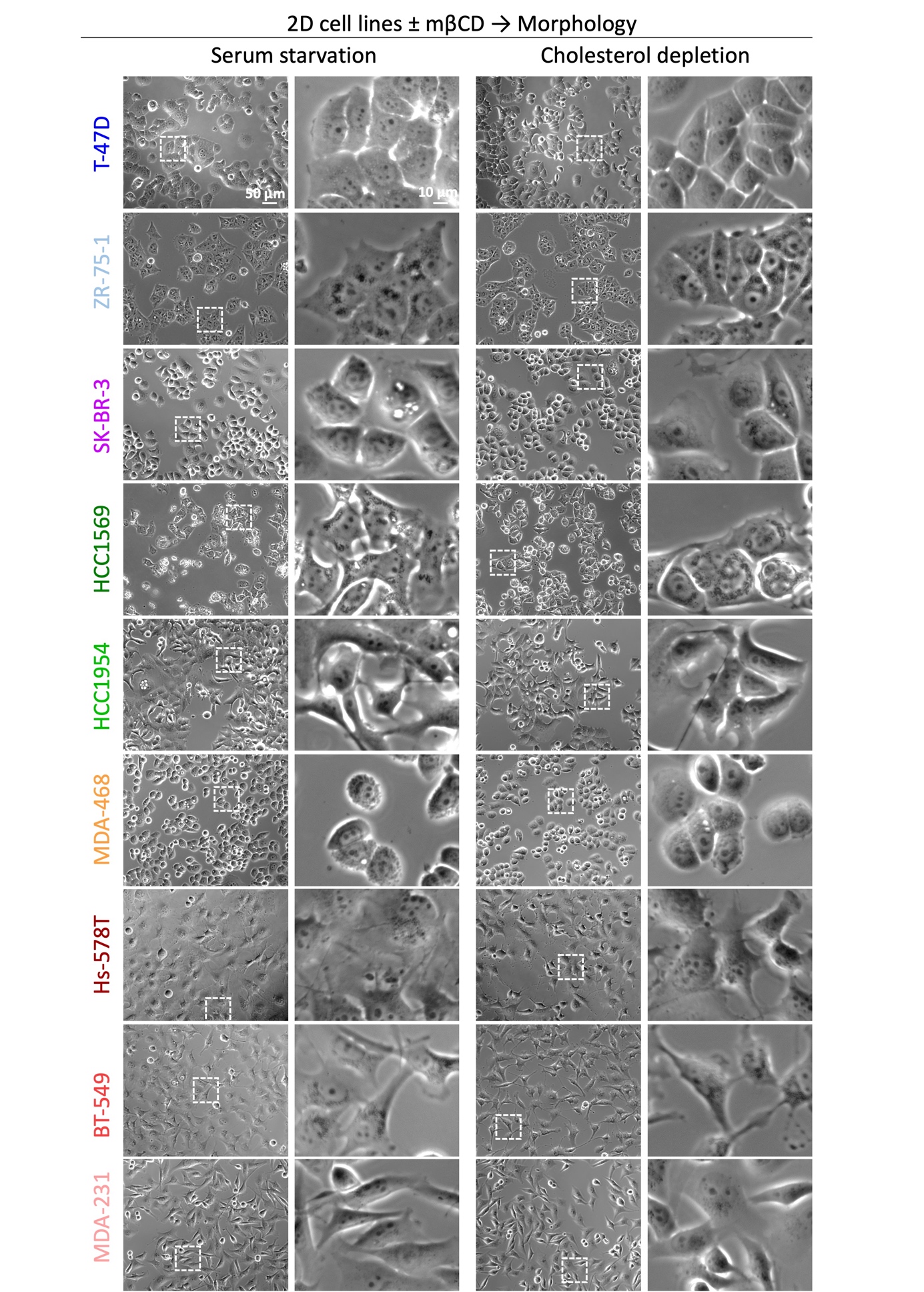
Figure S2. Cholesterol depletion does not affect 2D morphology of the 9 breast cancer cell lines.** The 9 cell lines were plated on fibronectin-coated coverslips, serum-starved combined or not with 2 mM mβCD (chol depletion) for 2 h then visualized by phase contrast microscopy. Insets better show cell morphology (n=1).

**
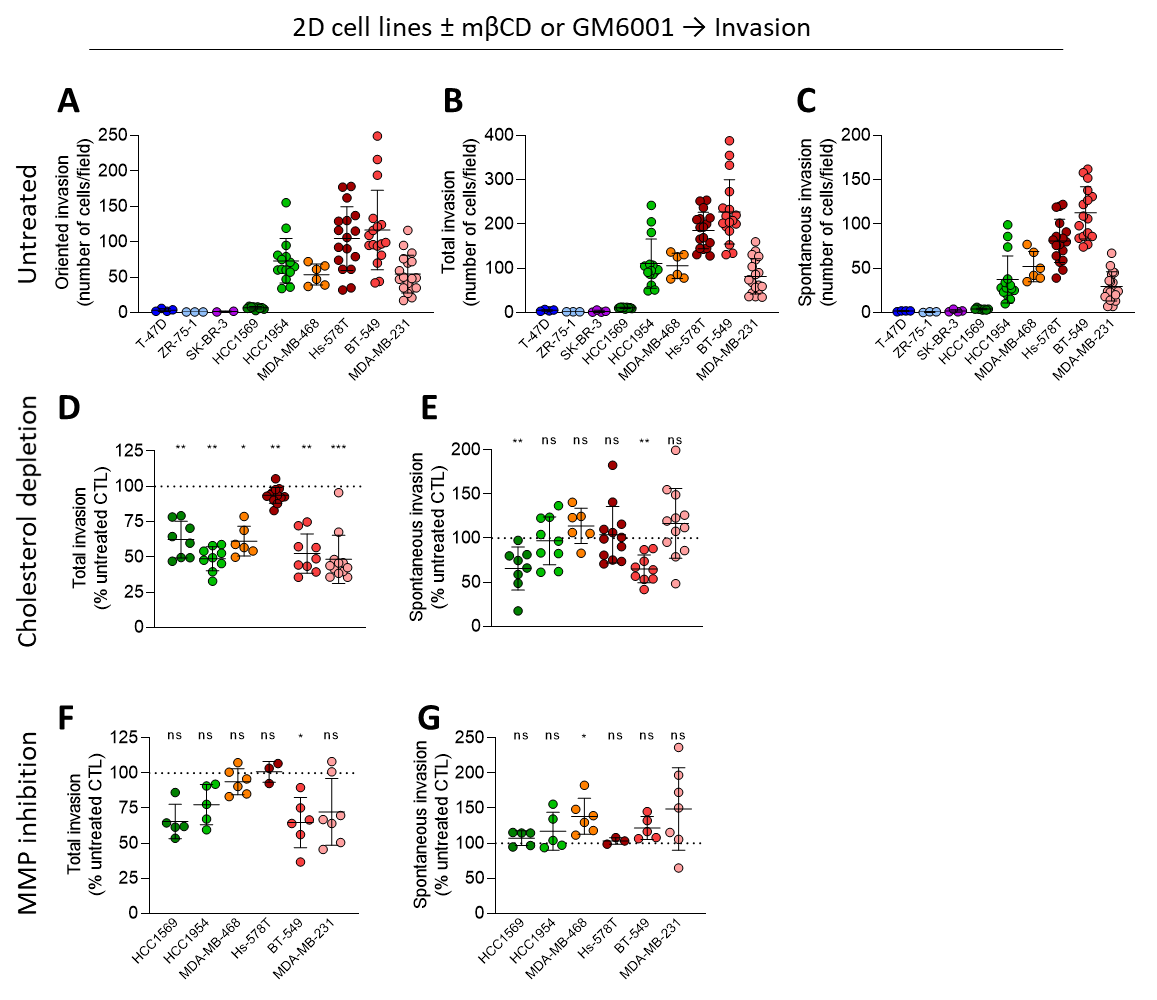
Figure S3. The 9 cell lines differ by the extent of invasion and cholesterol depletion but not MMP inhibition reduces spontaneous invasion in 2D of 2 cell lines.** Cell lines were serum-starved combined or not with 2 mM mβCD (chol depletion) for 2 h or treated with 10 μM GM6001 (MMP inhibition) during the whole experiment, and then tested for 2D cell invasion. Quantification of oriented (**A**), total (**B,D,F**) and spontaneous (**C,E,G**) 2D invasion of cell lines untreated (**A-C**) or treated with mβCD (**D,E**) or GM6001 (**F,G**) in Matrigel-coated Transwell toward 10% (total) or 0% (spontaneous) serum for 12 h. Oriented invasion was obtained by subtracting the spontaneous from the total number of invading cells. Data in A-C are expressed as number of untreated invading cells per field (n=2-18 Transwell from 1-7 independent experiments). Data in D-G are expressed as percentage of the number of untreated invading cells (n=6-12 Transwell from 2-5 independent experiments [chol depletion] and n=3-7 Transwell from 1-3 independent experiments [MMP inhibition]). Wilcoxon signed-rank test (D-G).

**
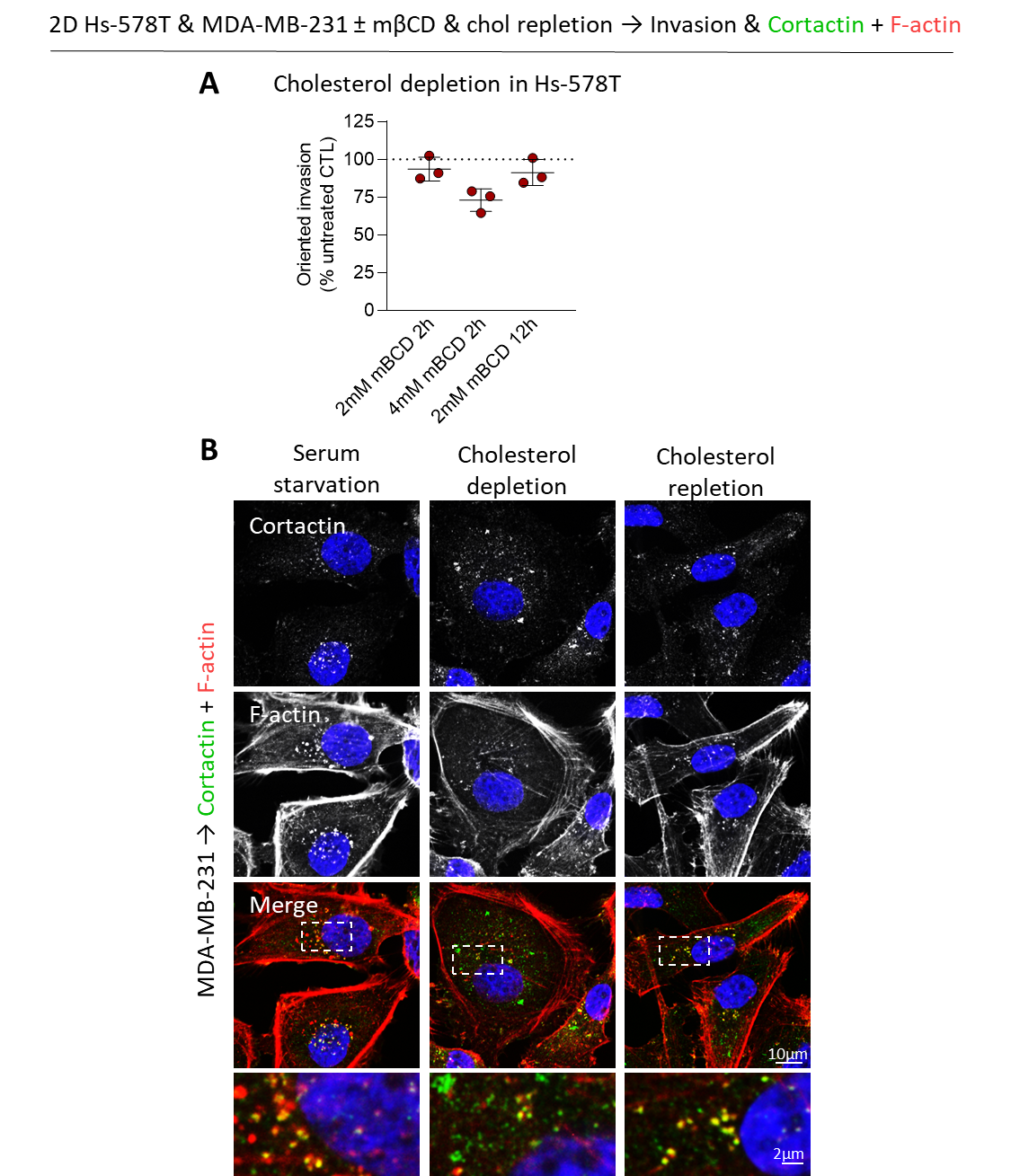
Figure S4. Cholesterol depletion only slightly decreases Hs-578T invasion in 2D and reversibly affects invadopodia formation in MDA-MB-231, as shown by cholesterol repletion.** (**A**) Quantification of 2D invasion in Matrigel-coated Transwell toward 10% serum for 12 h of Hs-578T serum-starved combined or not with 2 or 4 mM mβCD for 2 h, or with 2 mM mβCD during the whole experiment (n=3 Transwell from 1 experiment). (**B**) Confocal images of MDA-MB-231 plated on Oregon Green gelatin-coated coverslips, serum-starved combined or not with 2 mM mβCD (chol depletion) for 2 h, then incubated in serum-free medium containing 2 mM mβCD charged with chol (6:1 molar ratio; chol repletion) for 1 h and stimulated for 12 h with serum-containing medium. Cells were (immuno)labeled with anti-Cortactin, Phalloidin (F-actin) and Hoechst (nuclei). Insets show cortactin and F-actin colocalization at invadopodia (n=1).

**
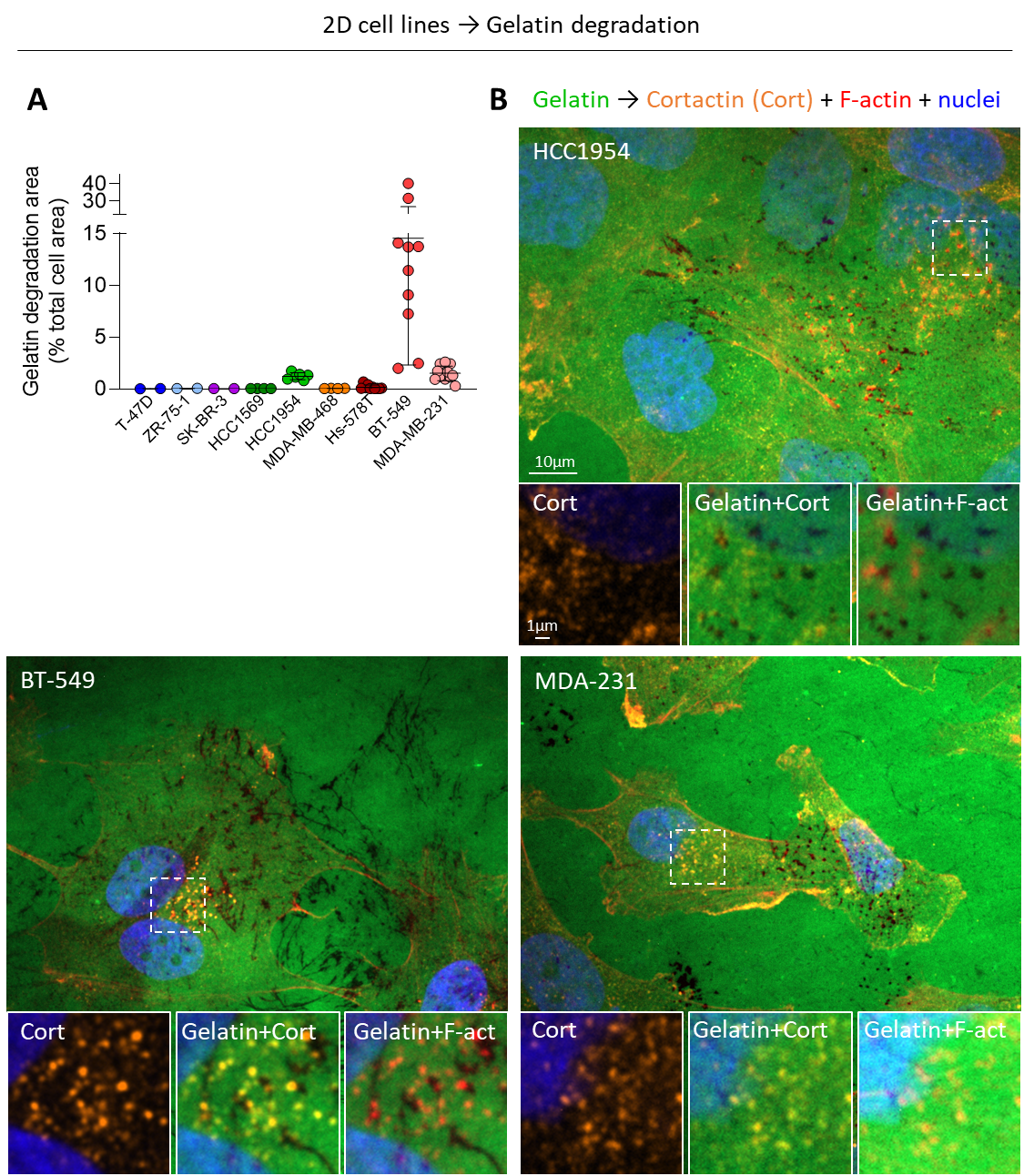
Figure S5. Only three cell lines are able to degrade the gelatin.** Cell lines were plated on Oregon Green gelatin-coated coverslips, serum-starved for 2 h, then stimulated for 6-12 h with serum-containing medium and (immuno)labeled with anti-Cortactin, Phalloidin (F-actin) and Hoechst (nuclei) as in Fig. 1I. (**A**) Quantification of gelatin degradation areas. Data are from 152-571 cells analyzed from n=11-82 images from 1-4 independent experiments. (**B**) Representative confocal images of gelatin degradation areas (black areas) and invadopodia of the 3 gelatin-degrading cell lines. For the 6 other cell lines, please refer to Fig. 1I.

**
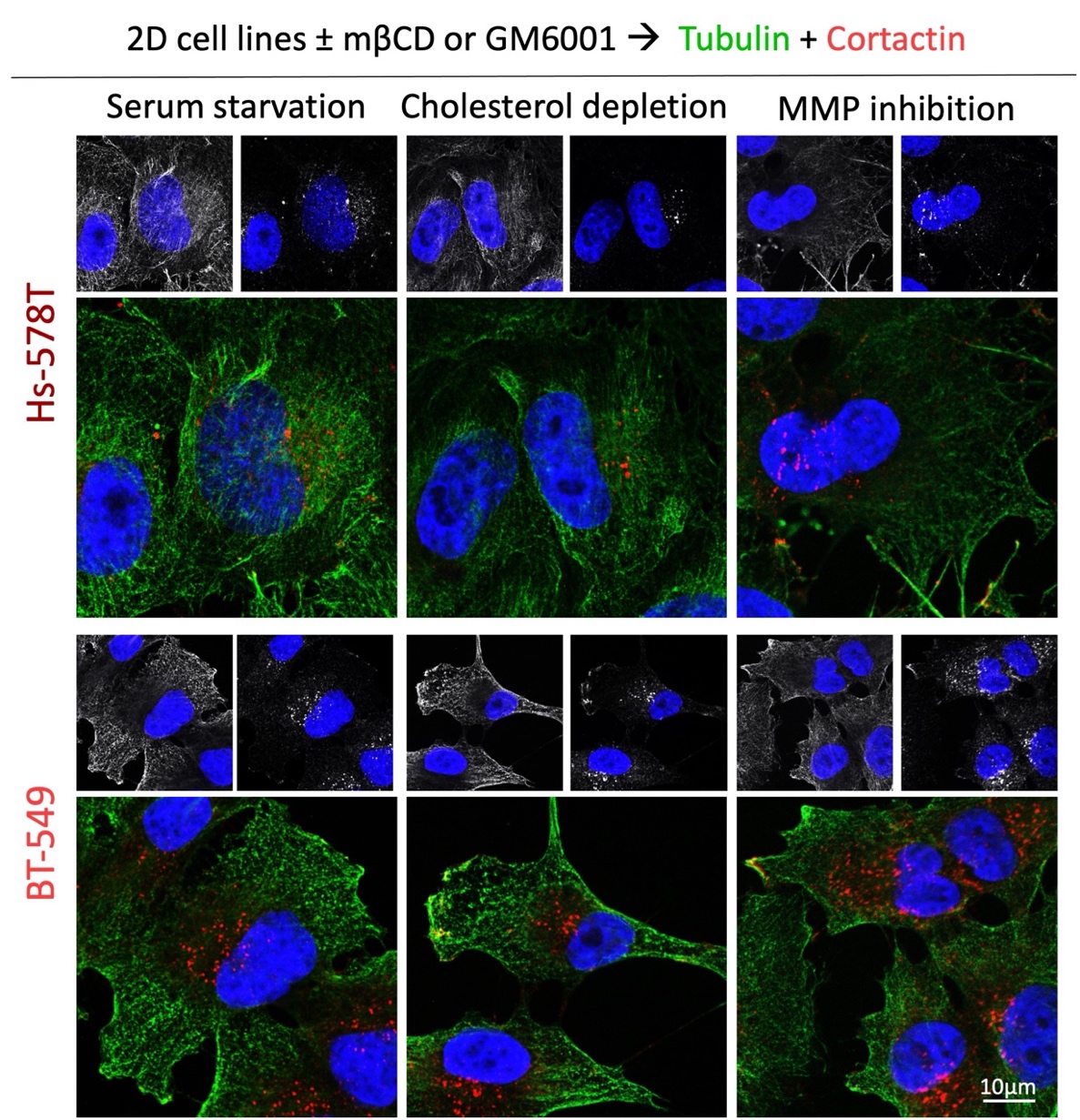
**

**Figure S6. Cholesterol depletion does not affect the distribution of microtubules in Hs-578T and BT-549 cell lines.** Hs-578T and BT-549 cells were plated on fibronectin-coated coverslips, serum-starved combined or not with 2 mM mβCD (chol depletion) for 2 h then stimulated for 4 h with serum-containing or 10 µM GM6001-supplemented medium (MMP inhibition). Cells were finally (immuno)labeled with anti-Cortactin (red), anti-α-Tubulin (microtubules, green) and Hoechst (nuclei) (n=1).

**
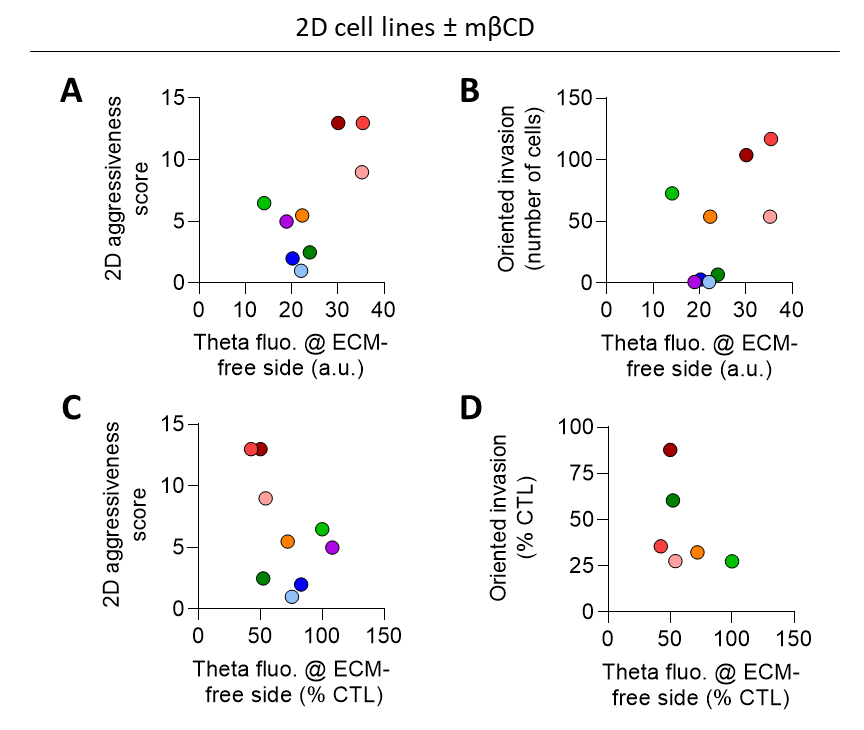
**

**Figure S7. Cholesterol-enriched domains at ECM-free side do correlate neither with breast cancer cell aggressiveness nor with invasion potential.** Relations between mean Theta fluorescence intensity at ECM-free side of each cell line treated or not (**A,B**) with 2 mM mβCD for 2 h (**C,D**), and 2D aggressiveness score (**A,C**) or oriented invasion potential (**B,D**).

**
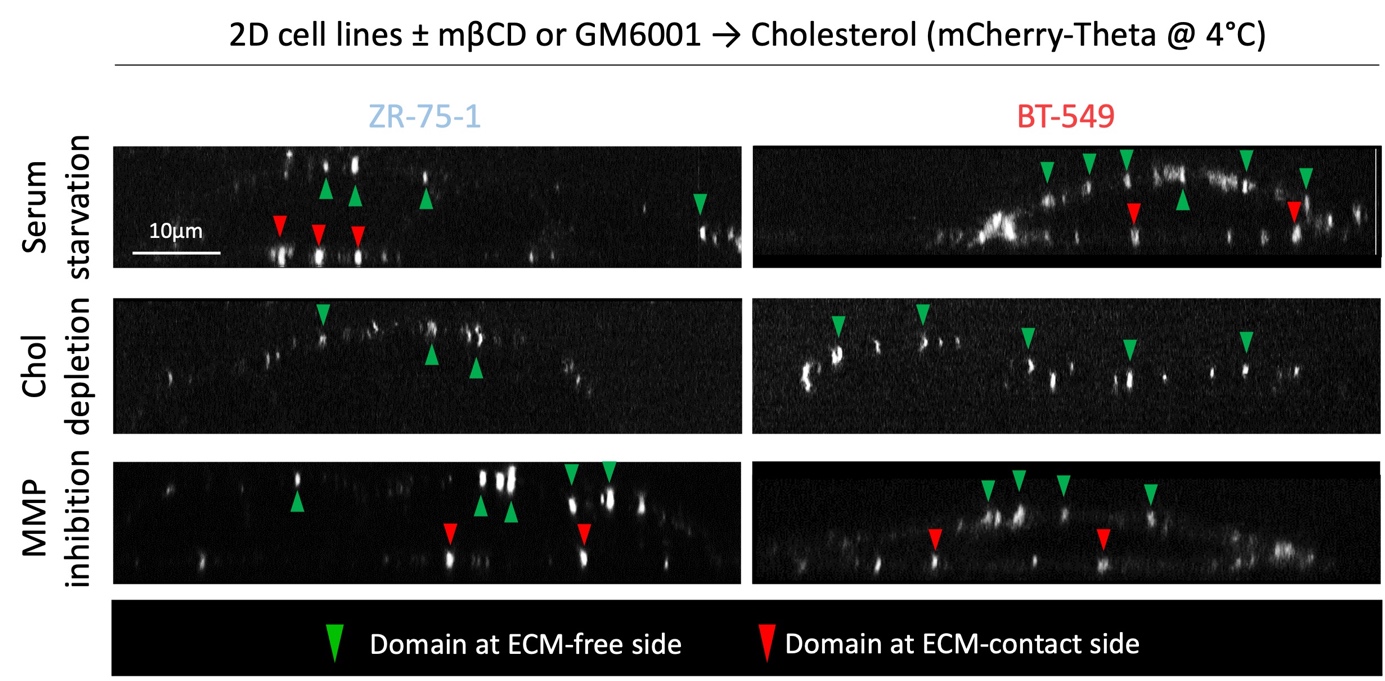
Figure S8. Cholesterol depletion, but not MMP inhibition, impairs cholesterol-enriched domains at the ECM-contact side in ZR-75-1 and BT-549 cells.** X-Z reconstructions of confocal images of ZR-75-1 and BT-549 cells plated on fibronectin-coated coverslips, treated or not (serum starvation) with 2 mM mβCD (chol depletion) or 10 μM GM6001 (MMP inhibition) for 2 h and then labeled at 4°C with the mCherry-Theta toxin fragment. Arrowheads show chol-enriched domains at ECM-free (green) and ECM-contact sides (red) (n=1).

**
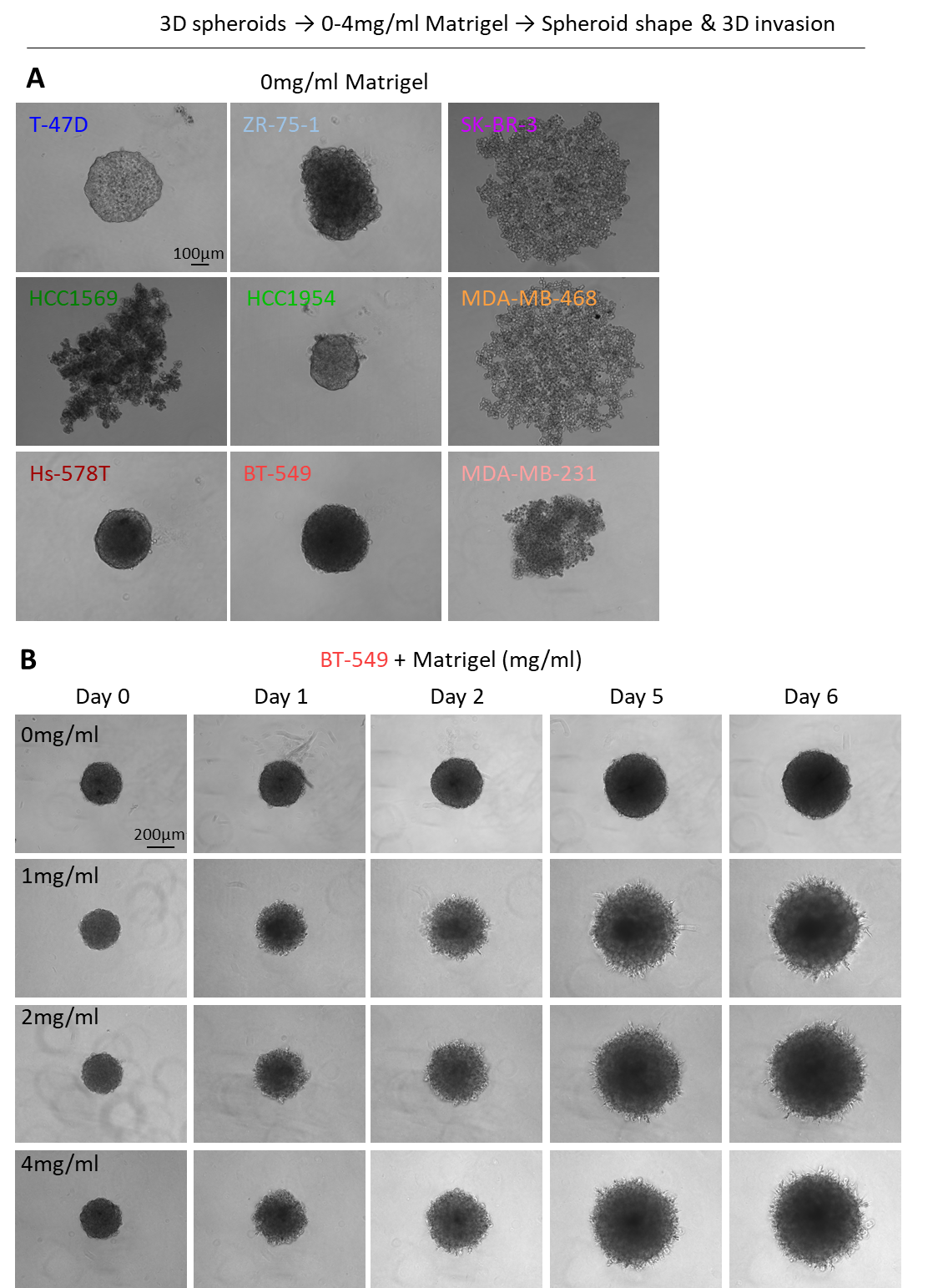
Figure S9. Differential breast cancer cell line spheroid shape and 3D invasion in Matrigel.** The 9 breast cancer cell lines were grown in ultra-low-attachment plates and then tested for spheroid formation (**A**). BT-549 spheroids were also tested for 3D Matrigel invasion (**B**). Representative phase contrast images of 9 breast cancer cell lines after 2 days of growth (**A**) or BT-549 spheroid 3D invasion over 1 week in the indicated Matrigel concentrations (**B**).

**
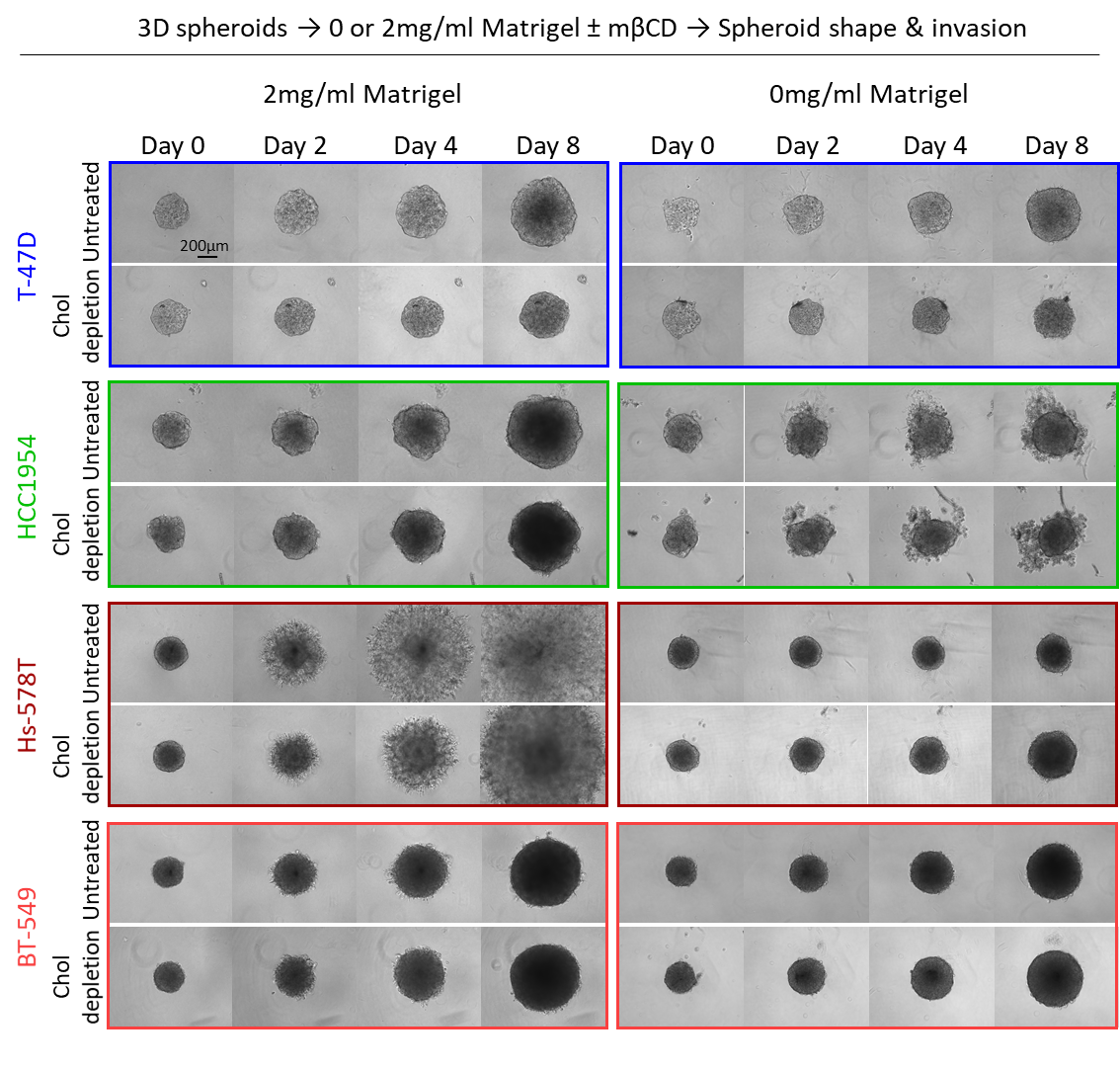
Figure S10. Cholesterol depletion reduces spheroid invasion but not growth.** T-47D, HCC1954, Hs-578T and BT-549 spheroids were grown for 3 days in ultra-low attachment plates and then embedded or not in 2 mg/mL Matrigel supplemented or not with 1.25 mM mβCD and allowed to grow for 8 days. Representative phase contrast images of spheroids treated or not with mβCD at days 0, 2, 4 and 8.

**
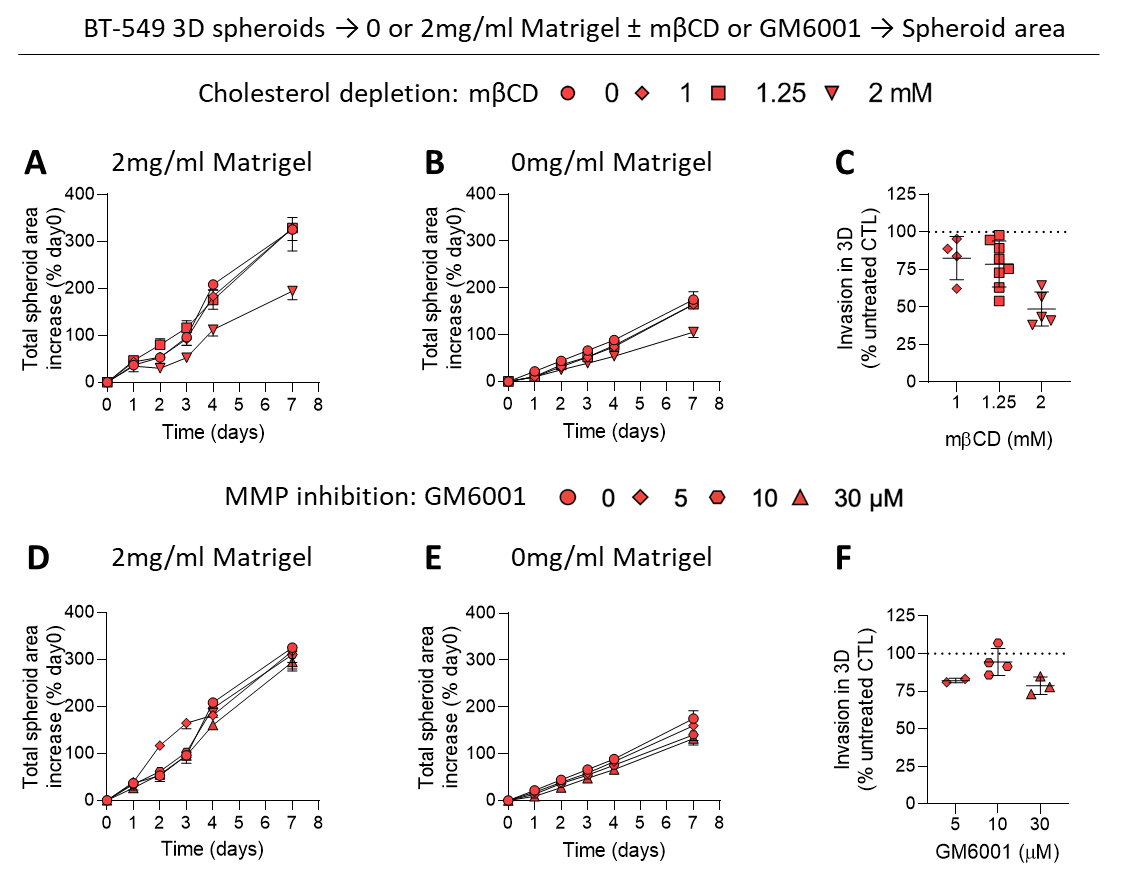
Figure S11. Cholesterol depletion, but not MMP inhibition, induces a dose-dependent decrease of BT-549 spheroid invasion.** BT-549 spheroids were grown for 3 days in ultra-low-attachment plates and then embedded (**A,D**) or not (**B,E**) in 2 mg/mL Matrigel supplemented or not (circle) with the indicated concentration of mβCD (chol depletion) or GM6001 (MMP inhibition) and allowed to grow for 7 days. (**A,B,D,E**) Time-course quantification of total spheroid area increase of the spheroids embedded (**A,D**) or not (**B,E**) in Matrigel and treated or not with mβCD or GM6001. Data are expressed as percent increase of day 0. Graphs are representative of 1 experiment with 2-5 spheroids per condition. (**C,F**) Quantification of invasion in 3D at day 4 of analysis of BT-549 treated with mβCD (**C**) or GM6001 (**F**). 3D invasion potential was obtained by subtracting the spheroid area increase without Matrigel from the area increase with Matrigel. Each data point represents 1 spheroid (in total 2-8 spheroids from 2 independent experiments).
